# Breast cancer screening needs assessment in 19 Northern California counties: geography, poverty, and racial/ethnic identity composition

**DOI:** 10.1007/s10552-024-01943-8

**Published:** 2024-12-01

**Authors:** Brittany L. Morgan Bustamante, Diana Miglioretti, Theresa Keegan, Eric Stewart, Anshu Shrestha, Nuen Tsang Yang, Rosemary D. Cress, Luis Carvajal-Carmona, Julie Dang, Laura Fejerman

**Affiliations:** 1 University of California Berkeley, Berkely, CA USA; 2 University of California Davis, Davis, CA USA; 3 Kaiser Permanente Washington Health Research Institute, Seattle, WA USA; 4 Cancer Registry of Greater California, Public Health Institute, Sacramento, CA USA

**Keywords:** Breast cancer, Needs assessment, Cancer center catchment area, Hispanic/Latino, Stage at diagnosis

## Abstract

**Purpose:**

To describe the area-level rate of breast cancers, the percentage of early-stage diagnoses (stage I-IIa), and associations between area-level measures of poverty, racial/ethnic composition, primary care shortage, and urban/rural/frontier status for the UC Davis Comprehensive Cancer Center (UCDCCC) catchment area.

**Methods:**

Using data from the SEER Cancer Registry of Greater California (2014–2018) and the California Department of Health Care Access and Information Medical Service Study Area, we conducted an ecological study in the UCDCCC catchment area to identify geographies that need screening interventions and their demographic characteristics.

**Results:**

The higher the percentage of the population identifying as Hispanic/Latino/Latinx, and the higher the percentage of the population below the 100% poverty level, the lower the odds of being diagnosed at an early-stage (OR = 0.98, 95% CI 0.96–0.99 and OR = 0.96, 95% CI 0.93–0.99, respectively). The association with poverty level was attenuated in the multivariable model when the Hispanic/Latino/Latinx population percentage was added. Several California counties had high poverty levels and differences in cancer stage distribution between racial/ethnic category groups. For all individuals combined, 65% was the lowest proportion of early-stage diagnoses for any geography. However, when stratified by racial/ethnic category, 11 geographies were below 65% for Hispanic/Latino/Latinx individuals, six for non-Hispanic Asian and Pacific Islander individuals, and seven for non-Hispanic African American/Black individuals, in contrast to one for non-Hispanic White individuals.

**Conclusions:**

Areas with lower percentages of breast cancers diagnosed at an early-stage were characterized by high levels of poverty. Variation in the proportion of early-stage diagnosis was also observed by race/ethnicity where the proportion of Hispanic/Latino/Latinx individuals was associated with fewer early-stage diagnoses.

**Impact:**

Results will inform the implementation of the UCDCCC mobile cancer prevention and early detection program, providing specific locations and populations to prioritize for tailored outreach, education, and screening.

**Supplementary Information:**

The online version contains supplementary material available at 10.1007/s10552-024-01943-8.

## Introduction

Breast cancer is the most common cancer among women in the United States and the second leading cause of cancer death [1, 2]. Disparities in breast cancer stage at diagnosis and outcomes by US Census racial and ethnic categories have been previously described, with individuals in the non-Hispanic/Latina-White category being more likely to be diagnosed with localized disease and to have a lower breast cancer mortality compared to Hispanic/Latina and African American/Black individuals [3–5].

The stage at diagnosis can be impacted by screening behavior, time to follow up of an abnormal mammogram, or the intrinsic biology of the tumor [4, 6]. Individuals who reside in rural areas or encounter other barriers to health care access and use (e.g., lack of health insurance, limited English proficiency, family responsibilities) are less likely to be up to date with their mammography screening [4, 6]. Additionally, individuals who live in poverty or identify with racial and ethnic minority categories often experience a longer time to follow up on abnormal mammography results [7, 8]. Finally, certain breast cancer subtypes are more aggressive and more likely to be diagnosed at an advanced stage [9].

The National Cancer Institute Designated Cancer Centers (NCIDCC) have a mandate to serve the population in the catchment area independently of their health care insurance status and ability to pay [10, 11]. Cancer Centers are charged with the responsibility of reducing or eliminating cancer health disparities in their region of influence [10, 11]. To enhance its response to this mandate, the UC Davis Comprehensive Cancer Center (UCDCCC) is planning to launch a new mobile cancer control program to take cancer prevention and diagnosis services to the most vulnerable communities in its catchment area. A key component of this program will be a mobile cancer prevention and early detection van serving underserved and rural regions where the population is most affected by structural or social barriers to health care. The planning phase of this new program requires a geographical and sociodemographic assessment of need: which specific locations include individuals who are not receiving standard of care for cancer prevention, and what economic, cultural, language, and educational barriers will need to be addressed? To start answering these questions, we conducted an ecological study of the 19 counties in the UCDCCC catchment area, assessing the association between the stage of breast cancer diagnosis and geography, poverty, and racial/ethnic category distribution at the level of the medical service study area (MSSA). MSSAs are smaller scale units than the county, providing a more precise geographic delineation for a mobile mammography program.

## Materials/methods

MSSAs are sub-city and sub-county geographic units created by the California Department of Health Care Access and Information (HCAI) to designate health professional shortage areas, medically underserved areas, or medically underserved populations. All California census tracts are assigned to an MSSA, and MSSAs do not cross county lines. MSSAs are developed to align with the social construct of “communities” in the sense of geographic, cultural, and sociodemographic similarities. Boundaries reflect community and neighborhood delineations [12].

For each MSSA, the number of females with breast cancer, by American Joint Committee on Cancer 7th edition anatomic stage at diagnosis (early: 0-IIa; vs. advanced: IIb +) [13, 14] and racial/ethnic census category (non-Hispanic White, non-Hispanic African American/Black, non-Hispanic Asian and Pacific Islander, and Hispanic/Latinx) were obtained from the SEER Cancer Registry of Greater California (2014 to 2018). The percentage of the population from different racial/ethnic categories, the percentage of the population below the 100% federal poverty level, rural, urban, and frontier area indicators, and primary care shortage area indicators, using the HCAI definition of a population to primary care physician ratio of 3,500:1 or 3,000:1 where the population demonstrates high need, were obtained from the HCAI MSSA dataset, which is derived from the 2010 decennial census [15]. HCAI definitions of rural (11 to < 250 people per square mile) and frontier (< 11 people per square mile) were used. An estimate of the female population aged 40 years or older for each MSSA was obtained from the US Census Bureau 2010 decennial census.

The spatial distribution of breast cancer burden across MSSAs was visualized using choropleth maps and a Jenks optimization method, which identifies groups with similar values and maximizes the difference between them, generated using the tmap package in R (RRID: SCR_000687) [16] for three outcomes: total number of breast cancer diagnoses, number of breast cancer diagnoses per 10,000 females age 40 and older, and percentage of breast cancer diagnoses that were early vs. advanced stage. Ecological associations were tested using logistic regression with early stage at diagnosis as the outcome (binomial random variable of the number of early-stage diagnoses out of the total number of diagnoses in the MSSA). Firstly, univariable models were tested to identify factors associated with the percentage of early-stage diagnosis. The following variables were included in the univariable logistic regression models: rural, frontier, and primary care shortage area binary indicator variables, and continuous variables for the percentage below the 100% federal poverty level, percentage of residents identifying as non-Hispanic White, percentage identifying as Hispanic/Latino/Latinx, percentage identifying as non-Hispanic Asian and Pacific Islander, and percentage identifying as non-Hispanic African American/Black. The multivariable logistic regression model included variables associated with early-stage diagnosis at a *p*-value of ≤ 0.05 (i.e., the percentage below the 100% federal poverty level, the percentage Hispanic/Latino/Latinx, and the primary care shortage area indicator). Results are presented as odds ratios (OR) and associated 95% confidence intervals (CI). The presence of spatial autocorrelation was assessed using global Moran’s I. We ran spatial error and spatial lag models using linear regression and compared our results to an ordinary least squares model using the Lagrange multiplier test.

Ethics approval was not required for this non-interventional ecological study as we utilized retrospective, anonymized surveillance data containing no private health information and involving no human subjects.

## Results

**Fig. 1:**
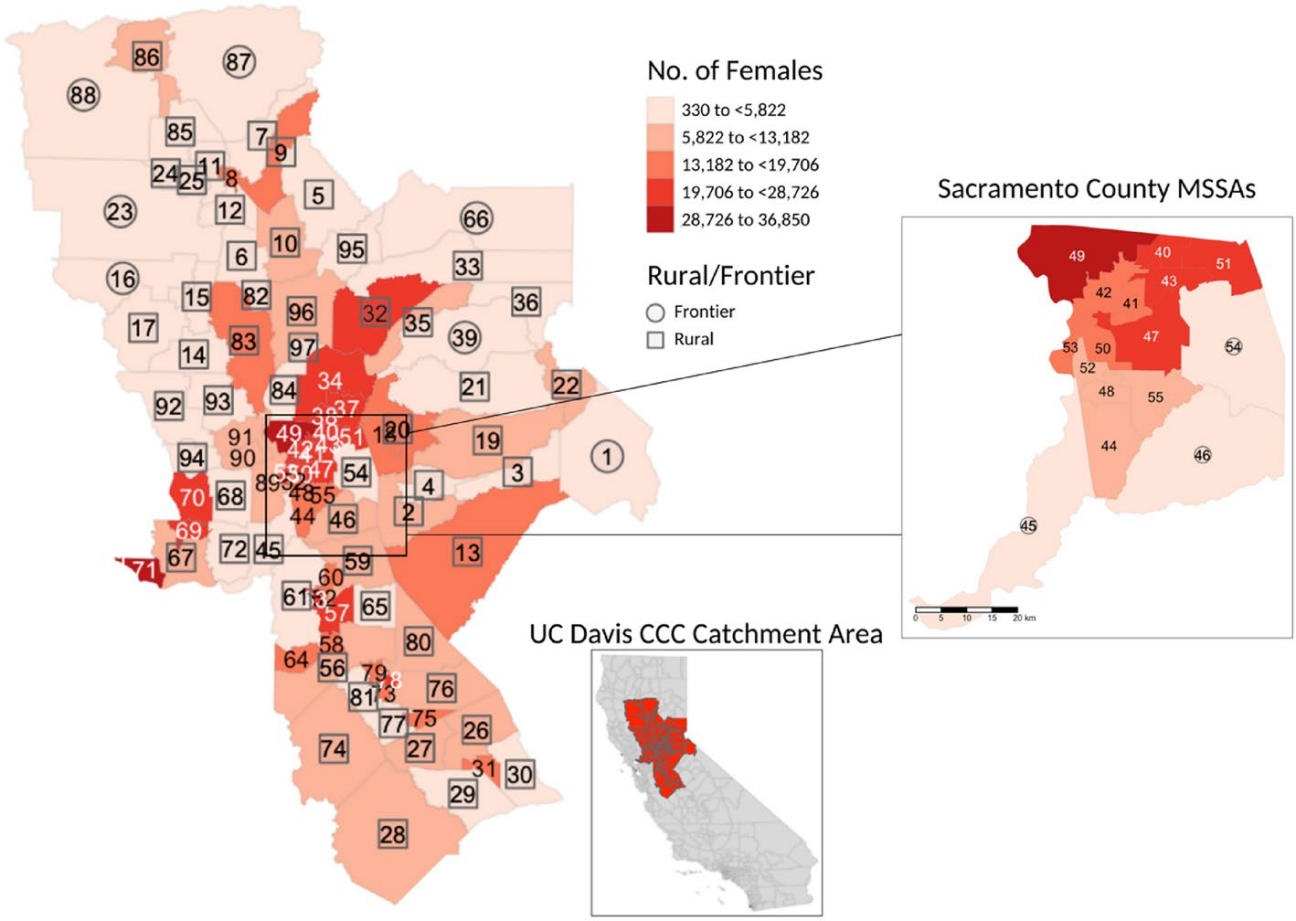
Distribution of the number of females aged 40 years or older across Medical Service Study Areas (MSSAs) in the UC Davis Comprehensive Cancer Center catchment area by rural/frontier status; pop out showing Sacramento County MSSAs; the names associated with each MSSA identification number can be found in Supplemental Table S1

The UCDCCC catchment area comprises 19 counties and 97 MSSAs. Of these, 35 were classified as urban, 55 as rural, and seven as frontier. The number of females aged 40 years or older varied from 330 to 36,850 across the MSSAs, which reflects the heterogeneity in population number within the catchment area, with the largest population size in the Sacramento County MSSAs (Fig. 1).

**Fig. 2:**
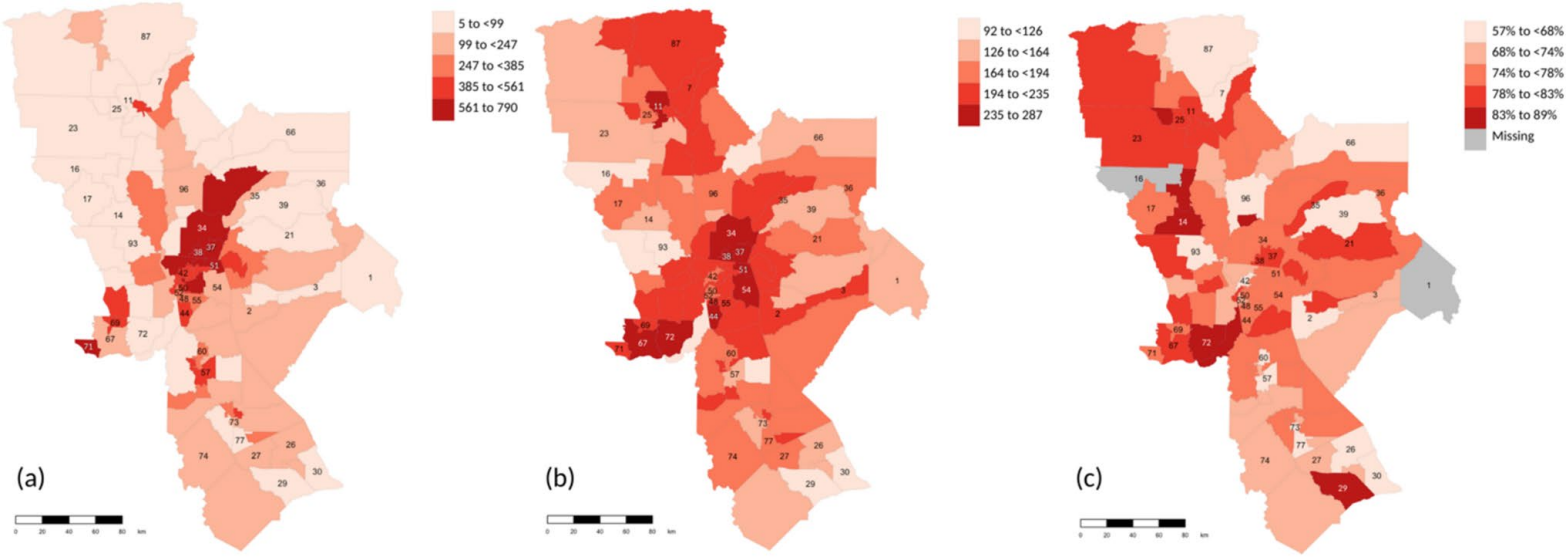
Distribution of the number of breast cancer diagnoses in each MSSA in the UC Davis Comprehensive Cancer Center catchment area **a** the rate of cancer diagnoses per 10,000 females aged 40 or older in each MSSA **b** and the percentage of early-stage diagnoses in each MSSA **c**; the names associated with each MSSA identification number can be found in Supplemental Table S1.To avoid number crowding in the figure, we added IDs to MSSAs within counties discussed in the results section

Overall, most breast cancer cases are concentrated in urban MSSAs in Sacramento County (Fig. 2a, MSSAs 40–55), with few other counties having MSSAs with large number of cases such as Placer (MSSAs 34–38) and Solano (MSSAs 67–71). Analyses of the breast cancer burden show high rates of breast cancer diagnoses per 10,000 females (> 235) in nine MSSAs within four counties (i.e., Butte, Placer, Sacramento, and Solano) (Fig. 2b). The lowest percentages of early-stage diagnoses (< 68%) were observed in thirteen MSSAs across 11 counties: Tehama, Butte, Sierra, Placer, Yuba, Yolo, Sacramento, Amador, San Joaquin, Stanislaus, and Merced (Fig. 2c; supplementary Table S1).

**Fig. 3:**
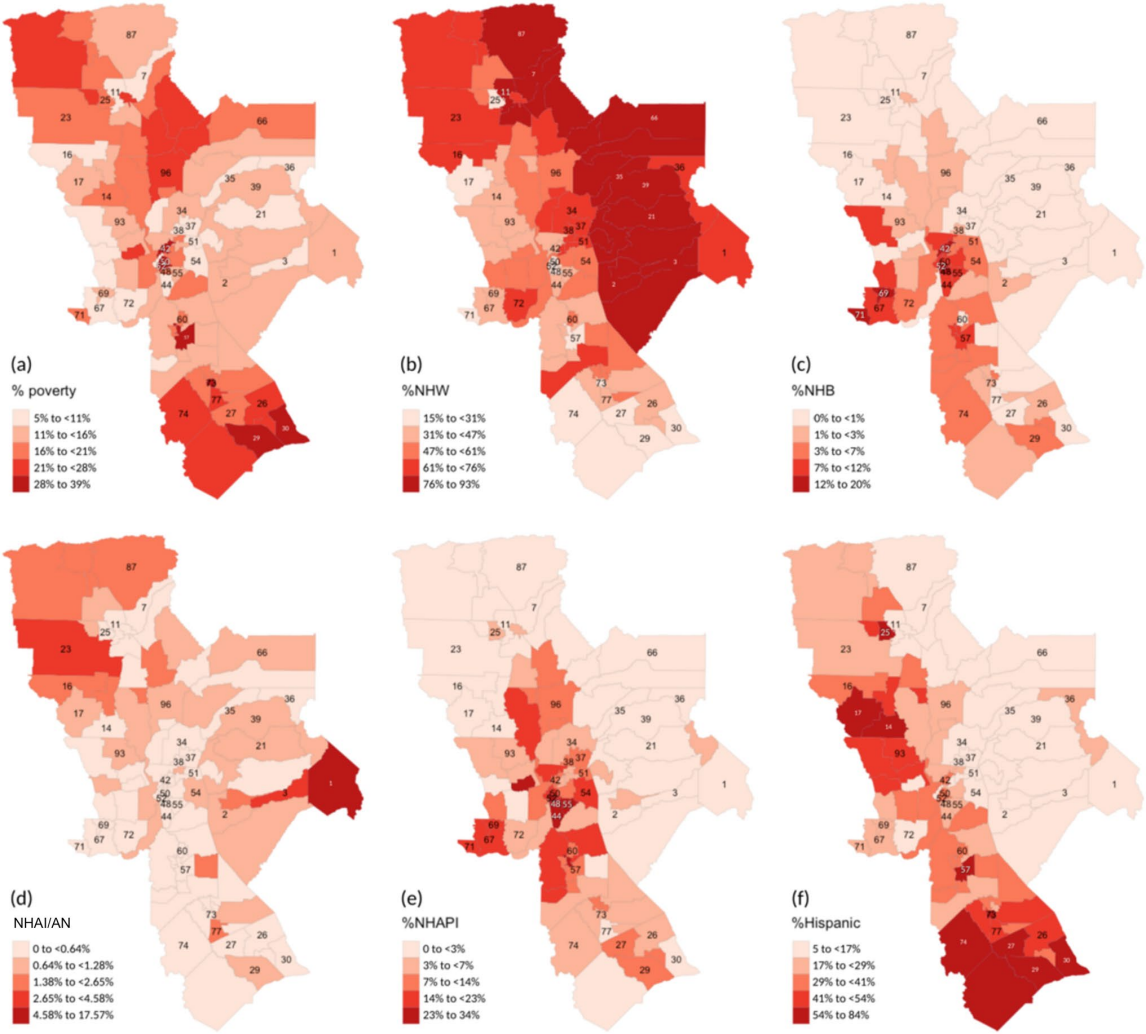
Percent of MSSA population living below the 100% federal poverty level **a** and percent of non-Hispanic White (NH-W) **b**, Hispanic/Latinx **c**, non-Hispanic Asian and Pacific Islander (NH-API) **d**, non-Hispanic African American/ Black (NH-B) **e**, and non-Hispanic American Indian and Alaska Native (NH-AI/AN) **f**; the names associated with each MSSA identification number can be found in Supplemental Table S1

The UCDCCC catchment area includes MSSAs with varying distributions of poverty and racial/ethnic category diversity (Fig. 3). The percentage of the MSSA population living below the 100% federal poverty level ranged from 5 and 11% (seven MSSAs) to 28–39% (seven MSSAs). Nine MSSAs included the highest percentage (54–84%) of the population identifying as Hispanic/Latino/Latinx, four MSSAs where the highest percentage (23–34%) of the population identifying as non-Hispanic Asian and Pacific Islander, three MSSAs with the highest percentage (12–20%) of the population identifying as non-Hispanic African American/Black, and one MSSAs with the highest percentage (< 4.58%) of the population identifying as non-Hispanic American Indian/Alaskan Native. One MSSA had a high percentage identifying as non-Hispanic American Indian/Alaskan Native (5–18%), but no available data on breast cancer stage at diagnosis due to the small number of individuals residing within the MSSA.

For all MSSAs, we compared the percentage of breast cancers diagnosed at an early-stage by racial/ethnic categories and by the percentage of the population below the 100% federal poverty level, identifying multiple MSSAs with important differences in the percentage of cases diagnosed at an early-stage (Table 1). Table 1 only includes MSSAs with more than 5,000 females > 39 years of age for which at least one racial/ethnic category has a percentage early-stage diagnosis of < 65% which is the lowest % early-stage diagnosis for any MSSA when considering all individuals without stratification by racial/ethnic category (see leftsupplementary data Table S1 including all MSSAs). Eleven MSSAs had a percent early-stage diagnosis of < 65% for Hispanic/Latino/Latinx individuals, six for non-Hispanic Asian and Pacific Islander individuals, and seven for non-Hispanic African American/Black individuals–compared to only one MSSA for non-Hispanic White individuals. Differences in the percentage of early-stage diagnosis by racial/ethnic category are notable for multiple MSSAs. As an example, in the Capitol Park/Del Paso Heights/Downtown/Gardenland/North Sacramento MSSA in Sacramento County, 50% of diagnoses occurred at early-stage for non-Hispanic Asian and Pacific Islander individuals, while in the other racial/ethnic categories, the percentages are higher, at 72%, 66%, and 68% in non-Hispanic White individuals, non-Hispanic African American/Black and Hispanic/Latinx individuals, respectively. Another example is the Antelope/Citrus Heights/Foothill Farms MSSA in Sacramento County, where the percent of cases diagnosed at an early stage among Hispanic/Latinx individuals is 59%, while for other racial/ethnic categories, the percentage is 75 to 80%. In El Dorado County, 59% of diagnoses were early-stage in Hispanic/Latinx individuals, compared to more than 75% in non-Hispanic White and non-Hispanic Asian and Pacific Islander individuals.

**Table 1:** Medical Service Study Areas (MSSAs) with more than 5,000 females aged ≥ 40 in the UC Davis Comprehensive Cancer Center Catchment Area with at least one racial/ethnic category population with < 65%^**‡**^ of breast cancers diagnosed at an early stage

County/MSSA (MSSA ID)	Female Pop. Aged ≥ 40 Years	Percent Below 100% Federal Poverty Level	Percentage of cases diagnosed at an early stage^‡^			
			NH-White	NH-Black	NH-API	Hispanic/Latino/ Latinx
Sacramento County						
Antelope/Citrus Heights/Foothill Farms (40)	27,859	19.0	78.4	76.7	80.0	59.3
Capitol Park/Del Paso Heights/Downtown/Gardenland/North Sacramento (42)	23,318	35.4	72.4	66.2	50.0	68.3
Carmichael/Fair Oaks/Gold River (43)	27,020	14.3	72.6	58.3	81.1	86.8
Central South Elk Grove/Franklin (44)	17,555	8.3	78.7	81.8	74.8	63.9
Clay/Galt/Herald/Wilton (46)	8600	18.0	83.5	N/A	83.3	64.9
Elverta/Natomas/North Highlands/Rio Linda (49)	36,850	14.6	75.4	63.2	69.0	74.3
Freeport/Meadowview (52)	17,225	29.6	80.0	68.3	64.3	76.4
Greenhaven/Land Park/Midtown/Pocket/ Riverside/Sutterville-Sacramento County (53)	23,790	10.0	81.5	57.5	77.9	80.4
Vineyard (55)	16,912	11.8	78.0	81.5	77.8	57.7
Merced County						
Ballico/Cressey/Delhi/Hilmar/Livingston (27)	7492	18.7	75.9	N/A	60.0	63.8
Merced Central and North/Merced Southeast (31)	14,767	25.2	72.4	60.0	61.5	71.4
San Joaquin County						
Banta/Escalon/Ripon/Vernalis (56)	11,828	11.4	72.8	62.5	77.8	65.8
French Camp/Stockton South/Stockton Southeast (57)	25,223	31.2	64.4	60.2	80.0	63.4
Lockeford/North Woodbridge (59)	8781	14.5	78.0	N/A	75.0	63.3
Lodi (60)	14,963	19.5	71.1	N/A	46.4	67.4
Stanislaus County						
Ceres/Modesto South Central (73)	15,631	31.2	71.0	N/A	75.0	60.5
Modesto East (78)	21,581	16.2	74.6	82.4	59.0	66.3
Modesto West (79)	19,426	18.2	77.7	63.6	74.2	73.6
El Dorado County						
Cool/Diamond Springs/Latrobe/Pilot Hill/ Placerville (20)	15,211	12.3	78.4	N/A	77.8	59.1
Tehama County						
Gerber/Los Flores/Proberta/Red Bluff (86)	9309	16.9	72.9	N/A	N/A	58.8
Yuba County						
Marysville-Yuba County (96)	12,194	22.3	67.7	72.7	66.7	64.3

^*^FPL: Federal Poverty Level; *NH: Non-Hispanic/Latinx; *API: Asian and Pacific Islander; NA: not available due to small numbers; ^‡^Racial/ethnic category groups with < 65% of cases diagnosed at an early stage are bolded for each MSSA; MSSA IDs are identifying numbers for geographic reference in the maps

### Association between the percent of the MSSA population below the 100% federal poverty level and the percentage of early-stage breast cancer diagnosis

In unadjusted (or crude) models, we observed that for a 10% increase in the percent of residents living below the 100% federal poverty level in the MSSA, the odds of early-stage diagnosis decreased by 0.04 (model 1, OR = 0.96, 95% CI 0.93–0.99) (Table 2). For a 10% increase in the percentage of non-Hispanic White residents in the MSSA, we observed a 0.01 increase in the odds of early-stage diagnosis (model 2, OR = 1.01, 95% CI 1.00–1.02). For a 10% increase in the percentage of Hispanic/Latino/Latinx residents in the MSSA, we observed a 0.02 decrease in the odds of early-stage diagnosis (model 3, OR = 0.98, 95% CI 0.96–0.99). MSSAs that are defined as primary care shortage areas have 0.95 times the odds of early-stage diagnosis compared to non-shortage areas (model 7, 95% CI 0.91–1.00). In a multivariable model (model 8) including percent poverty, percent Hispanic/Latino/Latinx residents, and a primary care shortage area indicator, the odds ratio for the percent Hispanic/Latino/Latinx residents did not change. However, the *p*-value changed to borderline statistical significance (OR = 0.98, 95% CI 0.97–1.00). We found no evidence of spatial autocorrelation in the outcome among the MSSAs (see supplementary data file S2).

**Table 2:** Univariable and multivariable logistic regression results measuring the association between the percentage of breast cancers diagnosed at an early-stage and area-level measures

	Model							
Area-level measures	(1) OR (95% CI)	(2) OR (95% CI)	(3) OR (95% CI)	(4) OR (95% CI)	(5) OR (95% CI)	(6) OR (95% CI)	(7) OR (95% CI)	(8) OR (95% CI)
Percentage poverty (per 10% change)	0.96^***^ (0.93, 0.99)							0.98 (0.94, 1.02)
Percentage NH-white (per 10% change)		1.01** (1.00, 1.02)						
Percentage Hispanic/Latinx			0.98^***^ (0.96, 0.99)					0.98* (0.97, 1.00)
Percentage NH-API (per 10% change)				0.99 (0.97, 1.02)				
Percentage NH-AA/B (per 10% change)					0.98 (0.95, 1.02)			
Rural vs. Urban						1.01 (0.95, 1.07)		
Frontier vs. Urban						0.98 (0.74, 1.31)		
Primary care shortage area (yes vs. no)							0.95** (0.91, 1.00)	0.99 (0.93, 1.04)
*Statistical significance levels				^*^p < 0.1	^**^p < 0.05	^***^p < 0.01		

*OR: odds ratio; CI: confidence interval; NH: non-Hispanic; API: Asian and Pacific Islander; AA/B: African American/Black*

## Discussion

Results of this study point to specific MSSAs in Sacramento, Merced, San Joaquin, Stanislaus, Tehama, El Dorado, and Yuba Counties, which have high levels of poverty and marked differences in stage at diagnosis by racial/ethnic category. These results will inform the implementation of the UCDCCC mobile cancer prevention and early detection program, prioritizing specific locations and populations to serve with tailored outreach, education materials, and screening. For example, in Antelope/Citrus Heights/Foothill Farms MSSA in Sacramento County, the percentage of cases diagnosed at an early stage among Hispanic/Latino/Latinx individuals is 59%, while for other racial/ethnic categories, the percentage is higher than 75%. Hence, our mobile mammography program in this area will be prepared to provide culturally tailored educational materials in English and Spanish to address potential language barriers. Additionally, several MSSAs had low proportion of diagnosis at an early stage for non-Hispanic African American/Black and non-Hispanic Asian and Pacific Islander individuals, which will require culturally tailored educational materials in multiple additional languages. These efforts will require early engagement of local community-based organizations trusted by community members as active partners. Similar geographic analyses have been conducted by few others[17–20] helping identify areas where existing services and infrastructure may be insufficient and where additional resource allocation may have the biggest impact on reducing cancer-related health disparities.

Our unadjusted bivariate analyses of the UCDCCC catchment area identified poverty, percentage of non-Hispanic White residents, percentage of Hispanic/Latino/Latinx residents, and primary care shortage areas to be associated with early-stage diagnosis at the MSSA-level. Contrary to what has been described in some national and regional analyses in the US [21–23], the rurality/frontier status of the MSSA was not significantly associated with the stage at diagnosis. The association between breast cancer screening behavior and geography has not been consistently reported in the literature, and a meta-analysis did not identify rurality as a factor contributing to lower screening attendance [24]. In the context of the UCDCCC catchment area, many of the rural and frontier MSSAs have lower percent of individuals under the 100% federal poverty limit compared to urban MSSAs with higher percent of individuals under the 100% federal poverty limit. Hence, in the UCDCCC catchment area, rurality might be overcome by lower barriers to screening based on income for a large number of MSSAs. In multivariable analysis, only the percentage of Hispanic/Latino/Latinx residents had a borderline significant association with the stage at diagnosis.

Access and transportation barriers to screening services that are dependent on technology, such as mammography for breast cancer screening or low-dose CT imaging for lung cancer screening, are important mechanisms explaining disparities in screening rates affecting rural and low-income communities [7]. Mobile services that bring the technology to under-resourced communities remove those barriers and increase screening rates [25–27]. Identifying geographic disparities in cancer health is essential in developing and tailoring mobile screening programs [14, 17–20, 28]. Previous studies have assessed the geographic distribution of breast cancer stage at diagnosis, screening rates, or screening access, including spatial analyses in smaller-than-county definitions to inform outreach programs or expansion of mammography facility locations to address disparities [14, 17–20, 28, 29]. Results highlight the inverse association between distance to a screening facility, adherence to screening recommendations, and the role of socioeconomic status, insurance status, and primary care physicians to population ratio [14, 17–20, 28].

Additional methods for identifying small areas to focus cancer control interventions with consideration of neighborhood-level factors and composite metrics of multiple breast cancer outcomes as an overall measure of burden could provide further refinement than the MSSA area scale selected for this project and could be implemented in future analyses [29]. Our analysis did not identify any geographic hotspots of breast cancer stage at diagnosis among MSSAs. However, others have identified geographic clustering of breast cancer cases among census tracts [30]. It is possible that while MSSAs provide more granularity than county-level analyses, there may still be heterogeneity within MSSAs that we are unable to capture at this level. Partnering with Federally Qualified Healthcare Centers to better understand and identify the needs of their unique patient populations or conducting community outreach surveys may complement and enhance the work done at larger geographic scales and help identify hotspots of underserved or missed neighborhoods that would benefit from more intensive interventions [31]. Notwithstanding, results showed that poverty and racial/ethnic makeup as well as disparate early-stage diagnoses by race/ethnicity within the same geographic area should be explored in other cancer center’s catchment areas as they may point to areas in need of mobile cancer prevention services that would not be identified without appropriate stratification.

There are limitations to ecological studies, including the lack of individual information on potential confounders like socioeconomic status or healthcare access. Additionally, MSSA boundaries are developed and redrawn with each decennial census. The most recent MSSA data is based on the 2010 census. To maintain consistency, we inferred the underlying female population size of each MSSA based on the 2010 census. The number of breast cancer cases comes from cancer registry data that has been collected between the years 2014 to 2018. This misalignment in the time period between the MSSA-level factors and the cancer registry data may have resulted in some measurement error. However, as exposures like neighborhood-level poverty and living in a primary care shortage area are influenced by larger, structural-level forces, it is unlikely major changes would have occurred within four years and influenced our conclusions.

Mobile mammography vans are widely used to address access barriers [32]. However, the wide implementation of mobile mammography programs by NCIDCC in the U.S. as an approach to addressing screening disparities in the catchment area is not yet standard practice. Systematic approaches to identify specific geographic locations and the demographic characteristics of populations that will benefit from the mobile mammography service are of utmost importance. This knowledge will facilitate the inclusion of supportive accessory materials to enhance service utilization, improve the client's experience, and maximize impact.

## Supplementary Information

Below is the link to the electronic supplementary material.

Supplementary file1 (HTML 4178 KB)

Supplementary file2 (XLSX 26 KB)

## Data Availability

The data supporting this study's findings are available from the SEER Cancer Registry of Greater California. Access to the data is granted by the management or data custodians through an application process. We have made the corresponding R code available in the supplemental material for replication.
