# Supplementary material for: Breast cancer screening needs assessment in 19 Northern California counties: geography, poverty, and racial/ethnic identity composition
Source: Cancer Causes Control. 2024 Dec 1;36(4):369–77. doi: 10.1007/s10552-024-01943-8 (PMC11982124; doi:10.1007/s10552-024-01943-8)
Supplement: Supplementary file 1 — Supplementary file1 (HTML 4178 KB) [file 10552_2024_1943_MOESM1_ESM.html]

Breast Cancer Stage at Diagnosis in 19 Northern California Counties: Geography, Poverty, and Racial/Ethnic Category Composition


# Breast Cancer Stage at Diagnosis in 19 Northern California Counties: Geography, Poverty, and Racial/Ethnic Category Composition

#### Brittany L Morgan Bustamante

#### 2024-05-08

## 1. Data Wrangling

```
# get population denominator
v10 <- load_variables(2010, "sf1", cache = TRUE)
view(v10)

tpop <- get_decennial(geography = "tract",
                state = "CA", 
                year = 2010, 
                variables = c(fem1 = "P012026", fem4 = "P012038",
                              fem5 = "P012039", fem6 = "P012040", fem7 = "P012041", 
                              fem8 = "P012042", fem9 = "P012043", fem10 = "P012044", 
                              fem11 = "P012045", fem12 = "P012046", fem13 = "P012047", 
                              fem14 = "P012048", fem15 = "P012049"),
                show_call = TRUE) %>%
        separate(NAME, c("Tract", "County", "State"), sep = ", ") %>%
        separate(Tract, c("Text1", "Text2", "tractc"), sep = " ") %>%
        spread(key = variable, value = value) %>%
        mutate(county = word(County , 1 , -2)) %>%
        mutate(fempop = (fem4 + fem5 + fem6 + fem7 + fem8 + fem9 + fem10 + fem11 + fem12 +
                           fem13 + fem14 + fem15), tract = as.numeric(tractc)) %>%
        select(tract, county, fempop)
```

```
## Getting data from the 2010 decennial Census
```

```
## Census API call: https://api.census.gov/data/2010/dec/sf1?get=P012026%2CP012038%2CP012039%2CP012040%2CP012041%2CP012042%2CP012043%2CP012044%2CP012045%2CP012046%2CP012047%2CP012048%2CP012049%2CNAME&for=tract%3A%2A&in=state%3A06
```

```
## Using Census Summary File 1
```

```
# fem2 = "P012036" (30-34), fem3 = "P012037" (35-39)

# attach to mssa tract data
mssa.tract <- mssa.tract %>%
              mutate(tract = as.numeric(TRACT), county = COUNTY, mssa = MSSA_NAME)
mssa.fem <- mssa.tract %>%
            left_join(tpop, by = c("county" = "county", "tract" = "tract"))

# dataset with only counties and variables of interest for mapping
study.mssa <- subset(mssa.fem, county == "Alpine" | county == "Amador" | county == "Butte" | 
                     county == "Calaveras" | county == "Colusa" | county == "El Dorado" | 
                     county == "Glenn" | county == "Merced" | county == "Nevada" | 
                     county == "Placer" | county == "Sacramento" | county == "San Joaquin" | 
                     county == "Sierra" | county == "Solano" | county == "Stanislaus" | 
                     county == "Sutter" | county == "Tehama" | county == "Yolo" | 
                     county == "Yuba") %>%
              mutate(mssa = MSSA_NAME) %>%
  group_by(mssa) %>%
          summarize(ftot = sum(fempop), tpop = sum(POP_CIV), povn = sum(POP100POV), 
                    tnhw = sum(NHS_WHITE), tnhb = sum(NHS_BLACK),
                    tnha = sum(NHS_ASIAN), tnhai = sum(NHS_AMIND_), 
                    nhisland = sum(NHS_ISLAND), hisp = sum(HISPANIC), 
                    sqmi = sum(AREA_SQMI), geometry = st_union(geometry)) %>%
          mutate(pct100pov = 100*(povn/tpop), pctnhw = 100*(tnhw/tpop), 
                 pctnhb = 100*(tnhb/tpop), 
                 pctnhapi = 100*((tnha + nhisland)/tpop), 
                 pctnhai = 100*(tnhai/tpop), pcthisp = 100*(hisp/tpop), 
                 popdens = tpop/sqmi) %>%
  ungroup() %>%
          select(mssa, ftot, pct100pov, popdens, pctnhw, pctnhb, pctnhapi, 
                 pctnhai, pcthisp, geometry)
  
# join to BC data, calculate case rate, include rural tag (OSHPD defines rural as population density less than 250 people per square mile, frontier as 11 people)
bc.mssa2 <- study.mssa %>%
          left_join(bc, by = "mssa") %>%
          mutate(n_rate = 10000*(n_cases/ftot), rural = case_when(popdens < 11 ~ 'Frontier',
                                                                  popdens < 250 ~ 'Rural'),
                 mssa.ID = as.character(mssa_ID))

# attach primary care shortage area indicator
pcsa <- pcsa %>%
        mutate(mssa = MSSA_NAME, pcsa = PCSA) %>%
        select(mssa, pcsa)
bc.mssa3 <- bc.mssa2 %>%
            left_join(pcsa, by = "mssa")

# save as data frame rather than tibble
bc.mssa = st_as_sf(as.data.frame(bc.mssa3))

# subset rural mssas
rural.mssa <- bc.mssa%>%
              drop_na(rural)

# california dataset for pop-out map 
ca.mssa2 <- mssa.fem %>%
          group_by(mssa) %>%
          summarize (ftot = sum(fempop), geometry = st_union(geometry), 
                     county = paste(unique(county))) %>%
          mutate(study = case_when(county == "Alpine" | county == "Amador" | 
                                   county == "Butte"  | county == "Calaveras" | 
                                   county == "Colusa" | county == "El Dorado" | 
                                   county == "Glenn"  | county == "Merced" | 
                                   county == "Nevada" | county == "Placer" | 
                                   county == "Sacramento" | county == "San Joaquin" | 
                                   county == "Sierra" | county == "Solano" | 
                                   county == "Stanislaus" | county == "Sutter" | 
                                   county == "Tehama" | county == "Yolo" | 
                                   county == "Yuba" ~ 1))

# sacramento dataset for zoom-in
sac.mssa <- subset(bc.mssa, county == "Sacramento")

# sacramento rural for zoom-in
sac.rural <- subset(sac.mssa, rural == "Rural")
  
# save as data frame rather than tibble
ca.mssa = st_as_sf(as.data.frame(ca.mssa2))
```

```
mssa_id <- subset(bc.mssa, mssa.ID %in% c(11, 34, 37, 38, 51, 54,
                                      44, 67, 72, 87, 7, 66,
                                      39, 96, 93, 42, 2, 60,
                                      57, 77, 26, 30, 16, 21,
                                      36, 35, 29, 73, 57, 42,
                                      50, 52, 27, 74, 17,
                                      14, 57, 25, 52, 48, 55,
                                      44, 69, 71, 23, 3, 1))
povmap <- subset(bc.mssa, mssa.ID %in% c(7, 16, 21, 36, 35, 67, 72, 30, 29, 73, 57, 
                                         41, 50, 52))
hispmap <- subset(bc.mssa, mssa.ID %in% c(30, 27, 29, 73, 17, 14, 57, 25))
apimap <- subset(bc.mssa, mssa.ID %in% c(52, 48, 55, 44))
aamap <- subset(bc.mssa, mssa.ID %in% c(48, 52, 69, 71))
aianmap <- subset(bc.mssa, mssa.ID %in% c(23, 3, 1))
```

## 2. Figures

## **Figure 1 - MSSA location, No. Females, Rural Indicator**

```
# figure one - mssa map with labels, CA map 
mssa_ca_map = tm_shape(bc.mssa) +
    tm_polygons(col = "ftot", style = "jenks", palette = "Reds", title = "No. of Females",
                border.alpha = 0.1, legend.show = TRUE) +
    tm_scale_bar(position = c("left", "bottom")) +
    tm_layout(main.title = "No. of Females Aged >39 Years per MSSA",
            main.title.size = 0.85, frame = FALSE,
            legend.format = list(digits = 0),
            legend.outside = TRUE, legend.outside.position = "right") +
  tm_text("mssa.ID", size = 0.6) +
    tm_shape(rural.mssa) + tm_symbols(size = 0.8, alpha = 0.1, shape = "rural", 
                                      title.shape = "Rural/Frontier") +
  tm_text("mssa_ID", size = 0.6)

ca_map = tm_shape(ca.mssa) + tm_polygons(border.alpha = 0.1) +
         tm_shape(bc.mssa) + tm_fill(col = "red") +
         tm_shape(bc.mssa) + tm_borders(lwd = 0.1)

sac_map = tm_shape(sac.mssa) +
  tm_polygons(col = "ftot", style = "jenks", palette = "Reds", title = "No. of Females",
              border.alpha = 0.1, legend.show = FALSE) +
  tm_scale_bar(position = c("left", "bottom")) +
  tm_text("mssa.ID", size = 0.85) +
  tm_shape(sac.rural) + tm_symbols(size = 1.2, alpha = 0.1, shape = "rural", 
                                   title.shape = "Rural/Frontier", legend.shape.show = FALSE) +
  tm_text("mssa_ID", size = 0.85)

# combine
mssa_ca_map
print(ca_map, vp = grid::viewport(0.8, 0.185, width = 0.2, height = 0.45))
```

## **Figure 2 - No. Diagnoses, Rate Map, Early Stage**

```
# mssa ID subset for mssas' referenced in manuscript
propmap <- subset(bc.mssa, mssa.ID %in% c(11, 34, 37, 38, 51, 54, 44, 67, 72))
earlymap <- subset(bc.mssa, mssa.ID %in% c(87, 7, 66, 39, 96, 93, 42, 2, 60, 
                                           57, 77, 26, 30))

# map no cases - overall
cases = tm_shape(bc.mssa) +
  tm_polygons(col = "n_cases", style = "jenks", palette = "Reds", 
              border.alpha = 0.1, title = "") +
  tm_scale_bar(position = c("left", "bottom")) +
    tm_layout(main.title = "Number of Breast Cancer Diagnoses per MSSA",
            main.title.size = 0.85, frame = FALSE, legend.format = list(digits = 0),
            legend.outside = TRUE, legend.outside.position = "right") 

# sac zoom no cases - overall
sac_cases = tm_shape(sac.mssa) +
  tm_polygons(col = "n_cases", style = "jenks", palette = "Reds", 
              border.alpha = 0.1, title = "", legend.show = FALSE)

# proportion map
proportion = tm_shape(bc.mssa) +
  tm_polygons(col = "n_rate", style = "jenks", palette = "Reds", 
              border.alpha = 0.1, title = "") +
  tm_scale_bar(position = c("left", "bottom")) +
    tm_layout(main.title = "Proportion of Cancer Patients in each MSSA among Women >39 Years Old \n (No. of cases per 10,000 females >39 years old)",
            main.title.size = 0.85, frame = FALSE, legend.format = list(digits = 0),
            legend.outside = TRUE, legend.outside.position = "right") +
  tm_shape(propmap) +
  tm_text("mssa_ID", size = 0.70)

# sac zoom proportion 
sac_proportion = tm_shape(sac.mssa) +
  tm_polygons(col = "n_rate", style = "jenks", palette = "Reds", 
              border.alpha = 0.1, title = "", legend.show = FALSE) +
  tm_text("mssa.ID", size = 0.7) +
  tm_scale_bar(position = c("left", "bottom"))

# early stage
early = tm_shape(bc.mssa) +
  tm_polygons(col = "early0iia", style = "jenks", palette = "Reds",
              border.alpha = 0.1, title = "",
              legend.format = list(fun = function(x) 
                paste0(formatC(x, digits = 0, format = "f"), "%"))) +
  tm_scale_bar(position = c("left", "bottom")) +
    tm_layout(main.title = "Percent of Diagnoses that are Early Stage Breast Cancer
                            per MSSA",
            main.title.size = 0.85, frame = FALSE, legend.format = list(digits = 0),
            legend.outside = TRUE, legend.outside.position = "right") +
  tm_shape(earlymap) +
  tm_text("mssa_ID", size = 0.70)

# sac zoom early
sac_early = tm_shape(sac.mssa) +
  tm_polygons(col = "early0iia", style = "jenks", palette = "Reds", 
              border.alpha = 0.1, title = "", legend.show = FALSE) +
  tm_text("mssa.ID", size = 0.7) +
  tm_scale_bar(position = c("left", "bottom"))

# combine
tmap_arrange(cases, proportion, early)
```

```
## Legend labels were too wide. The labels have been resized to 0.62, 0.47, 0.41, 0.41, 0.41. Increase legend.width (argument of tm_layout) to make the legend wider and therefore the labels larger.
```

```
## Legend labels were too wide. The labels have been resized to 0.47, 0.41, 0.41, 0.41, 0.41. Increase legend.width (argument of tm_layout) to make the legend wider and therefore the labels larger.
```

```
## Legend labels were too wide. The labels have been resized to 0.37, 0.37, 0.37, 0.37, 0.37, 0.57. Increase legend.width (argument of tm_layout) to make the legend wider and therefore the labels larger.
```

## **Figure 3 - Poverty Map**

```
# poverty
poverty = tm_shape(bc.mssa) +
  tm_polygons(col = "pct100pov", style = "jenks", palette = "Reds", 
              border.alpha = 0.1, title = "", 
              legend.format = list(fun = function(x) 
              paste0(formatC(x, digits = 0, format = "f"), "%"))) +
    tm_layout(frame = FALSE, legend.format = list(digits = 0),
            legend.outside = TRUE, legend.outside.position = "left") +
  tm_shape(povmap) +
  tm_text("mssa_ID", size = 0.40)
```

```
# sac zoom poverty
sac_poverty = tm_shape(sac.mssa) +
  tm_polygons(col = "pct100pov", style = "jenks", palette = "Reds", 
              border.alpha = 0.1, title = "", legend.show = FALSE) +
  tm_text("mssa.ID", size = 0.4)
```

## **Figure 4 - Race/Ethnicity Maps with Percent Poverty**

```
# non-Hispanic white
nhw = tm_shape(bc.mssa) +
  tm_polygons(col = "pctnhw", style = "jenks", palette = "Reds", 
              border.alpha = 0.1, title = "", 
              legend.format = list(fun = function(x) 
              paste0(formatC(x, digits = 0, format = "f"), "%"))) +
    tm_layout(frame = FALSE, legend.format = list(digits = 0),
            legend.outside = TRUE, legend.outside.position = "left")
# Hispanic
hisp = tm_shape(bc.mssa) +
  tm_polygons(col = "pcthisp", style = "jenks", palette = "Reds", 
              border.alpha = 0.1, title = "", 
              legend.format = list(fun = function(x) 
              paste0(formatC(x, digits = 0, format = "f"), "%"))) +
    tm_layout(frame = FALSE, legend.format = list(digits = 0),
            legend.outside = TRUE, legend.outside.position = "left") +
  tm_shape(hispmap) +
  tm_text("mssa_ID", size = 0.40)

# non-Hispanic Asian/Pacific Islander
api = tm_shape(bc.mssa) +
  tm_polygons(col = "pctnhapi", style = "jenks", palette = "Reds", 
              border.alpha = 0.1, title = "", 
              legend.format = list(fun = function(x) 
              paste0(formatC(x, digits = 0, format = "f"), "%"))) +
    tm_layout(frame = FALSE, legend.format = list(digits = 0),
            legend.outside = TRUE, legend.outside.position = "left") +
  tm_shape(apimap) +
  tm_text("mssa_ID", size = 0.40)

# non-Hispanic African-American/Black
aab = tm_shape(bc.mssa) +
  tm_polygons(col = "pctnhb", style = "jenks", palette = "Reds", 
              border.alpha = 0.1, title = "", 
              legend.format = list(fun = function(x) 
              paste0(formatC(x, digits = 0, format = "f"), "%"))) +
    tm_layout(frame = FALSE, legend.format = list(digits = 0),
            legend.outside = TRUE, legend.outside.position = "left") +
  tm_shape(aamap) +
  tm_text("mssa_ID", size = 0.40)

# non-Hispanic American Indian/Alaskan Native
aian = tm_shape(bc.mssa) +
  tm_polygons(col = "pctnhai", style = "jenks", palette = "Reds", 
              border.alpha = 0.1, title = "", 
              legend.format = list(fun = function(x) 
              paste0(formatC(x, digits = 0, format = "f"), "%"))) +
    tm_layout(frame = FALSE, legend.format = list(digits = 0),
            legend.outside = TRUE, legend.outside.position = "left") +
  tm_shape(aianmap) +
  tm_text("mssa_ID", size = 0.40)
```

```
fig3 <- tmap_arrange(poverty, nhw, hisp, api, aab, aian)

# tmap_save(fig3, "figure3.png", width = 1000, height = 750, dpi = 300)

fig3
```

```
## Legend labels were too wide. The labels have been resized to 0.41, 0.37, 0.37, 0.37, 0.37. Increase legend.width (argument of tm_layout) to make the legend wider and therefore the labels larger.
```

```
## Legend labels were too wide. The labels have been resized to 0.37, 0.37, 0.37, 0.37, 0.37. Increase legend.width (argument of tm_layout) to make the legend wider and therefore the labels larger.
```

```
## Legend labels were too wide. The labels have been resized to 0.41, 0.37, 0.37, 0.37, 0.37. Increase legend.width (argument of tm_layout) to make the legend wider and therefore the labels larger.
```

```
## Legend labels were too wide. The labels have been resized to 0.46, 0.46, 0.41, 0.37, 0.37. Increase legend.width (argument of tm_layout) to make the legend wider and therefore the labels larger.
```

```
## Legend labels were too wide. The labels have been resized to 0.46, 0.46, 0.46, 0.41, 0.37. Increase legend.width (argument of tm_layout) to make the legend wider and therefore the labels larger.
```

```
## Legend labels were too wide. The labels have been resized to 0.46, 0.46, 0.46, 0.46, 0.41. Increase legend.width (argument of tm_layout) to make the legend wider and therefore the labels larger.
```

```
# sac zoom race/ethnicity
sac_re = tm_shape(sac.mssa) +
  tm_fill(c("pct100pov", "pctnhw", "pctnhb", "pctnhai", "pctnhapi", "pcthisp"), 
          style = "jenks", 
          title = c("% poverty", "% non-Hispanic White", "% non-Hispanic Black", 
                    "% non-Hispanic \n American Indian",
                    "% non-Hispanic \n Asian/Pacific Islander", "% Hispanic"),
          legend.show = TRUE) +
  tm_borders("grey25", alpha = 0.1) +
  tm_text("mssa.ID", size = 0.7) +
  tm_layout(main.title = "",
            frame = FALSE, 
            legend.format = list(fun = function(x) 
            paste0(formatC(x, digits = 0, format = "f"), "%")), legend.width = 0.7, 
            legend.text.size = 0.6, legend.position = c("right", "bottom"))

sac_re
```

## **Figure 5 - PCSA Map**

```
# pcsa
pcsa = tm_shape(bc.mssa) +
  tm_polygons(col = "pcsa", style = "cat", palette = "Reds", 
              border.alpha = 0.1, title = "") +
  tm_scale_bar(position = c("left", "bottom")) +
    tm_layout(main.title = "Primary Care Shortage Areas",
            main.title.size = 0.85, frame = FALSE, legend.format = list(digits = 0),
            legend.outside = TRUE, legend.outside.position = "right") +
  tm_shape(mssa_id) +
  tm_text("mssa_ID", size = 0.60)

pcsa
```

```
# sac zoom pcsa
sac_pcsa = tm_shape(sac.mssa) +
  tm_polygons(col = "pcsa", style = "cat", palette = "Reds", 
              border.alpha = 0.1, title = "", legend.show = FALSE) +
  tm_text("mssa.ID", size = 0.6) +
  tm_scale_bar(position = c("left", "bottom"))

sac_pcsa
```

## **Figure 6 - Rural/Frontier Map**

```
# rural
rural = tm_shape(bc.mssa) +
    tm_polygons(col = "rural", style = "cat", palette = "Reds", 
              border.alpa = 0.1, title = "", textNA = "Urban") +
    tm_scale_bar(position = c("left", "bottom")) +
    tm_layout(main.title = "Rural and Frontier MSSAs",
            main.title.size = 0.85, frame = FALSE, legend.format = list(digits = 0),
            legend.outside = TRUE, legend.outside.position = "right") +
  tm_shape(mssa_id) +
  tm_text("mssa_ID", size = 0.60)

rural
```

```
# sac zoom rural
sac_rural = tm_shape(sac.mssa) +
    tm_polygons(col = "rural", style = "cat", palette = "Reds", 
              border.alpa = 0.1, title = "", textNA = "Urban") +
    tm_text("mssa.ID", size = 0.6) +
    tm_scale_bar(position = c("left", "bottom")) +
    tm_layout(frame = FALSE, legend.format = list(digits = 0),
            legend.outside = TRUE, legend.outside.position = "right")

sac_rural
```

## **% Early Table, 65% cutoff, female pop > 5,000**

```
# create smaller table as df - early by re (any group less than 65%)
race.eth65 <- as.data.frame(bc.mssa) %>%
          subset(ftot >= 5000) %>%
          subset((earlynhw > 0 & earlynhw < 65.00) | (earlynhb > 0 & earlynhb < 65.00) | 
                (earlyapi > 0 & earlyapi < 65.00) | (earlyhisp > 0 & earlyhisp < 65.00)) %>%
          select(mssa, mssa_ID, earlynhw, earlynhb, earlyapi, earlyhisp, pct100pov, ftot)
        

# remove re with no data
race.eth65 <- na.omit(race.eth65)
  
race.eth65
```

```
##                                                                  mssa mssa_ID
## 4                              Antelope/Citrus Heights/Foothill Farms      40
## 10                            Ballico/Cressey/Delhi/Hilmar/Livingston      27
## 11                                       Banta/Escalon/Ripon/Vernalis      56
## 21 Capitol Park/Del Paso Heights/Downtown/Gardenland/North Sacramento      42
## 22                                    Carmichael/Fair Oaks/Gold River      43
## 23                                   Central South Elk Grove/Franklin      44
## 27                                            Clay/Galt/Herald/Wilton      46
## 43                          Elverta/Natomas/North Highlands/Rio Linda      49
## 50                                                Freeport/Meadowview      52
## 51                      French Camp/Stockton South/Stockton Southeast      57
## 55          Greenhaven/Land Park/Midtown/Pocket/Riverside/Sutterville      53
## 64                                         Lockeford/North Woodbridge      59
## 68                                                         Marysville      96
## 69                          Merced Central and North/Merced Southeast      31
## 71                                                       Modesto East      78
## 72                                                       Modesto West      79
## 93                                                           Vineyard      55
##    earlynhw earlynhb earlyapi earlyhisp pct100pov  ftot
## 4     78.44    76.67    80.00     59.26 18.975439 27859
## 10    75.86     0.00    60.00     63.79 18.690807  7492
## 11    72.78    62.50    77.78     65.79 11.406452 11828
## 21    72.44    66.15    50.00     68.33 35.415103 23318
## 22    72.59    58.33    81.08     86.84 14.297190 27020
## 23    78.66    81.82    74.79     63.93  8.276855 17555
## 27    83.46   100.00    83.33     64.86 18.037534  8600
## 43    75.40    63.16    69.03     74.31 14.606631 36850
## 50    80.00    68.32    64.29     76.36 29.576677 17225
## 51    64.42    60.22    80.00     63.36 31.215759 25223
## 55    81.54    57.45    77.87     80.39  9.945221 23790
## 64    78.00   100.00    75.00     63.33 14.477912  8781
## 68    67.66    72.73    66.67     64.29 22.257024 12194
## 69    72.41    60.00    61.54     71.43 25.235555 14767
## 71    74.58    82.35    58.97     66.25 16.219509 21581
## 72    77.73    63.64    74.19     73.61 18.209644 19426
## 93    78.02    81.48    77.78     57.69 11.805476 16912
```

## **Supplement Table: % Early, All MSSAs**

```
early.all <- as.data.frame(bc.mssa) %>%
          select(mssa, mssa_ID, earlynhw, earlynhb, earlyapi, earlyhisp, early0iia,
                 pct100pov, ftot)
        
early.all
```

```
##                                                                                            mssa
## 1                    Alleghany/Calpine/Downieville/Goodyear Bar/Sattley/Sierra City/Sierraville
## 2                        Alpine Village/Bear Valley/Kirkwood/Markleeville/Mesa Valley/Woodfords
## 3  Angels Camp/Arnold/Copperopolis/Mountain Ranch/Murphys/San Andreas/Valley Springs/West Point
## 4                                                        Antelope/Citrus Heights/Foothill Farms
## 5                                                                               Arbuckle/Grimes
## 6                                                         Arcade/Arden/Town and Country Village
## 7                                    Artois/Elk Creek/Glenn/Grindstone Indian Rancheria/Willows
## 8                                                                       Atwater/Snelling/Winton
## 9                                                             Auburn/Newcastle/Lincoln/Sheridan
## 10                                                      Ballico/Cressey/Delhi/Hilmar/Livingston
## 11                                                                 Banta/Escalon/Ripon/Vernalis
## 12                                                             Benicia/Collinsville/Suisun City
## 13                                      Berry Creek/Brownsville/Concow/Feather Falls/Forbestown
## 14                                                                     Biggs/East Biggs/Gridley
## 15                                               Browns Valley/Brownsville/Dobbins/Oregon House
## 16                                         Bryte/Broderick/Clarksburg/Riverview/West Sacramento
## 17                                                          Butte Meadows/Cohasset/Forest Ranch
## 18                                                 Cameron Park/El Dorado Hills/Shingle Springs
## 19                                                                         Camino/Pollock Pines
## 20                                                                              Capay/Lake/Nord
## 21                           Capitol Park/Del Paso Heights/Downtown/Gardenland/North Sacramento
## 22                                                              Carmichael/Fair Oaks/Gold River
## 23                                                             Central South Elk Grove/Franklin
## 24                                                                  Ceres/Modesto South Central
## 25                                                                            Chapmantown/Chico
## 26                                                   Clarksburg/Hood/Isleton/Locke/Walnut Grove
## 27                                                                      Clay/Galt/Herald/Wilton
## 28                                                                          Colfax/Meadow Vista
## 29                                                                                       Colusa
## 30                                          Cool/Diamond Springs/Latrobe/Pilot Hill/Placerville
## 31                                                              Corning/Los Molinos/Tehama/Vina
## 32                                        Crows Landing/Empire/Grayson/Newman/Patterson/Westley
## 33                                                       Dairyville/Manton/Mineral/Paynes Creek
## 34                                                                                        Davis
## 35                                                                               Denair/Turlock
## 36                                                                                        Dixon
## 37                                    Dollar Point/Kings Beach/Sunnyside/Tahoe City/Tahoe Vista
## 38                                                                  Dos Palos/Gustine/Los Banos
## 39                                                                                       Durham
## 40                                    East Sacramento/La Riviera/Mather/Rancho Cordova/Rosemont
## 41                                                                     El Nido/Merced Southwest
## 42                                                        Elk Grove/Laguna/Laguna West/Lakeside
## 43                                                    Elverta/Natomas/North Highlands/Rio Linda
## 44                                                                               Esparto/Rumsey
## 45                                                                                    Fairfield
## 46                                                                            Farmington/Linden
## 47                                          Florin/Fruitridge/Oak Park/Parkway/South Sacramento
## 48                                                                            Folsom/Orangevale
## 49                                                                                   Foresthill
## 50                                                                          Freeport/Meadowview
## 51                                                French Camp/Stockton South/Stockton Southeast
## 52                                       Garden Valley/Georgetown/Greenwood/Kelsey/Volcanoville
## 53                                                         Gerber/Los Flores/Proberta/Red Bluff
## 54                                Grass Valley/Nevada City/Penn Valley/Rough and Ready/Wildwood
## 55                                    Greenhaven/Land Park/Midtown/Pocket/Riverside/Sutterville
## 56                                                                                Hamilton City
## 57                                                                                      Hughson
## 58                                                         Ione/Jackson/Pine Grove/Sutter Creek
## 59                                                                                        Keyes
## 60                                                                              Knights Landing
## 61                                                                              Lathrop/Manteca
## 62                                                                             Le Grand/Planada
## 63                                                                                     Live Oak
## 64                                                                   Lockeford/North Woodbridge
## 65                                                                                         Lodi
## 66                                                           Loduga/Maxwell/Princeton/Stonyford
## 67                                                               Magalia/Paradise/Stirling City
## 68                                                                                   Marysville
## 69                                                    Merced Central and North/Merced Southeast
## 70                                                                   Meridian/Robbins/Yuba City
## 71                                                                                 Modesto East
## 72                                                                                 Modesto West
## 73                                                                      Nicolaus/Pleasant Grove
## 74                                                      North Bloomfield/North San Juan/Truckee
## 75                                                                            Oakdale/Riverbank
## 76                                                                                       Orland
## 77                                                                  Oroville/Palermo/Thermalito
## 78                                                                       Paskenta/Rancho Tehama
## 79                                                                                      Pioneer
## 80                                                                                     Plymouth
## 81                                                                   Rancho Murieta/Sloughhouse
## 82                                                                                    Rio Vista
## 83                                                                          Rocklin/Granite Bay
## 84                                                                                    Roseville
## 85                                                                                       Salida
## 86                                                                             South Lake Tahoe
## 87                                                                    South Woodbridge/Thornton
## 88                                                    Stockton North Central/Stockton Northeast
## 89                                                             Stockton Northwest/Stockton West
## 90                                                                                        Tracy
## 91                                                                                    Vacaville
## 92                                                                                      Vallejo
## 93                                                                                     Vineyard
## 94                                                                                    Wheatland
## 95                                                                                     Williams
## 96                                                                                      Winters
## 97                                                                                     Woodland
##    mssa_ID earlynhw earlynhb earlyapi earlyhisp early0iia pct100pov  ftot
## 1       66    69.23       NA       NA        NA     66.67 16.287625  1062
## 2        1       NA       NA       NA        NA        NA 15.118243   330
## 3       13    69.95       NA   100.00     90.91     72.87 12.102300 14387
## 4       40    78.44    76.67    80.00     59.26     77.07 18.975439 27859
## 5       14    85.71       NA       NA     87.50     87.50 16.500192  1013
## 6       41    78.00    66.67    73.17     81.82     77.10 16.289652 22647
## 7       23    75.00       NA     0.00        NA     79.49 15.888107  2686
## 8       26    65.59       NA    75.00     67.65     66.43 23.405055  9646
## 9       34    77.87    80.00    77.42     78.85     77.67 11.630600 25467
## 10      27    75.86     0.00    60.00     63.79     69.92 18.690807  7492
## 11      56    72.78    62.50    77.78     65.79     70.64 11.406452 11828
## 12      67    81.05    85.71    84.06     82.35     82.35  6.362002  8148
## 13       5    75.00     0.00     0.00        NA     75.00 25.272980  1685
## 14       6    73.58       NA       NA     57.14     70.42 18.626603  3361
## 15      95    73.33     0.00     0.00        NA     70.59 25.087336  1517
## 16      89    69.74       NA    73.08     71.74     69.23 20.298480 11436
## 17       7    65.52     0.00     0.00        NA     67.74 10.231700  1519
## 18      18    83.85       NA    80.65     83.33     83.13  6.540214 17862
## 19      19    73.15     0.00       NA     83.33     73.73 11.145328  7548
## 20      11    83.78     0.00       NA        NA     82.22  9.269612  1621
## 21      42    72.44    66.15    50.00     68.33     68.50 35.415103 23318
## 22      43    72.59    58.33    81.08     86.84     74.15 14.297190 27020
## 23      44    78.66    81.82    74.79     63.93     76.43  8.276855 17555
## 24      73    70.97       NA    75.00     60.49     67.11 31.240203 15631
## 25       8    81.17       NA    75.00     68.75     78.94 24.336807 19210
## 26      45    87.50     0.00     0.00        NA     88.89 14.400572  1557
## 27      46    83.46   100.00    83.33     64.86     80.45 18.037534  8600
## 28      35    80.89     0.00       NA        NA     82.08  9.342965  8527
## 29      15    85.71       NA       NA     90.91     87.50 15.509409  1742
## 30      20    78.40       NA    77.78     59.09     76.60 12.268982 15211
## 31      85    74.19     0.00     0.00     77.78     74.32 20.783373  4232
## 32      74    73.44    83.33    66.67     72.73     72.26 22.151817  7109
## 33      87    64.29     0.00     0.00        NA     67.74 14.219625  1369
## 34      90    81.08       NA    66.67     68.18     78.62 27.728528 11870
## 35      75    71.69       NA    75.00     71.43     71.17 17.632831 16082
## 36      68    74.63       NA       NA     75.00     74.75 13.258272  4581
## 37      36    74.47     0.00       NA        NA     74.55 10.877056  3171
## 38      28    76.54       NA    66.67     72.46     72.94 26.002616 11082
## 39      12    80.00     0.00     0.00        NA     80.95 11.486732  1464
## 40      47    76.37    74.14    81.82     72.06     75.85 17.405699 28726
## 41      29    88.89     0.00       NA     81.82     84.85 38.801772  3561
## 42      48    80.00    68.85    81.18     76.79     76.92 18.351475 15911
## 43      49    75.40    63.16    69.03     74.31     72.41 14.606631 36850
## 44      92       NA       NA     0.00     83.33     83.33  9.916887  1245
## 45      69    79.59    81.51    73.86     70.42     77.43 14.296461 23816
## 46      65    76.92     0.00     0.00      0.00     76.92 13.159336  1293
## 47      50    75.00    67.74    70.91     76.84     74.04 31.664652 24461
## 48      51    78.27    80.00    77.27     73.02     77.51  5.954157 26925
## 49      39    68.00     0.00       NA        NA     66.67 12.898356  1906
## 50      52    80.00    68.32    64.29     76.36     72.16 29.576677 17225
## 51      57    64.42    60.22    80.00     63.36     65.85 31.215759 25223
## 52      21    79.25     0.00     0.00        NA     79.63  8.847241  3104
## 53      86    72.89       NA       NA     58.82     70.85 16.892953  9309
## 54      32    77.03       NA    85.71     76.47     77.18 12.484542 25718
## 55      53    81.54    57.45    77.87     80.39     78.63  9.945221 23790
## 56      25       NA     0.00     0.00    100.00     81.82 20.149853   662
## 57      76    76.61     0.00    85.71     76.09     75.69 20.080642  9697
## 58       2    71.21     0.00       NA        NA     67.11 13.635144  7433
## 59      77    64.71     0.00   100.00     57.14     66.07 26.028159  2967
## 60      93       NA       NA       NA        NA     66.67 14.970368  1040
## 61      58    71.98    66.67    77.78     70.45     71.63 12.066226 15955
## 62      30       NA     0.00     0.00     70.00     57.14 31.498698  1515
## 63      82    73.68     0.00       NA     72.73     73.53 19.216108  2329
## 64      59    78.00   100.00    75.00     63.33     75.14 14.477912  8781
## 65      60    71.12       NA    46.43     67.44     68.18 19.478530 14963
## 66      16       NA       NA       NA        NA        NA 10.118265   637
## 67       9    80.41     0.00       NA    100.00     81.25 17.411823 13511
## 68      96    67.66    72.73    66.67     64.29     67.87 22.257024 12194
## 69      31    72.41    60.00    61.54     71.43     69.12 25.235555 14767
## 70      83    75.56       NA    73.33     77.78     75.23 16.782544 18417
## 71      78    74.58    82.35    58.97     66.25     72.20 16.219509 21581
## 72      79    77.73    63.64    74.19     73.61     75.68 18.209644 19426
## 73      84    78.57     0.00     0.00      0.00     75.00  9.505259   697
## 74      33    75.00       NA       NA     62.50     74.73 12.295367  4959
## 75      80    79.07       NA       NA     77.42     78.28 15.002975 12661
## 76      24    92.59     0.00       NA     54.55     85.51 23.377627  3002
## 77      10    77.34       NA       NA     78.57     75.72 23.698211 12260
## 78      88    82.35     0.00     0.00        NA     83.33 26.987281  1313
## 79       3    75.68       NA     0.00        NA     69.77 10.772914  2050
## 80       4    81.82     0.00     0.00      0.00     81.82 12.663848  1573
## 81      54    79.80    55.56    84.21     60.00     77.70  5.195468  4902
## 82      72    88.06       NA   100.00        NA     86.75 10.937677  2897
## 83      37    80.20    71.43    85.71     93.48     81.28  6.113182 27997
## 84      38    81.75    69.23    77.14     75.38     80.25  8.892663 25917
## 85      81    76.09       NA       NA     73.33     77.91 16.640218  5284
## 86      22    75.49     0.00       NA     66.67     74.14 15.453760  7076
## 87      61    76.67       NA    76.92     78.95     74.75 13.935566  5822
## 88      62    74.07    70.21    65.22     69.14     69.92 29.200319 19706
## 89      63    72.22    74.19    85.29     75.86     75.12 16.995216 21447
## 90      64    75.29    69.05    77.91     65.43     72.47  9.095523 19523
## 91      70    81.06    75.76    81.13     66.67     78.95  9.146081 22591
## 92      71    78.42    69.18    79.55     73.13     76.42 16.408715 36543
## 93      55    78.02    81.48    77.78     57.69     76.14 11.805476 16912
## 94      97    83.33     0.00     0.00        NA     86.67 17.648527   967
## 95      17    68.75       NA       NA        NA     75.00 13.073969  1091
## 96      94    73.68     0.00     0.00     66.67     71.88  8.866348  1867
## 97      91    76.05     0.00    81.25     73.75     75.56 14.005124 13182
```

## **3. Regression Analysis**

1. Data Exploration - correlation matrix

```
library(corrplot)
```

```
## corrplot 0.92 loaded
```

```
# subset data (percent each race/ethnicity, percent poverty, case rate, percent early dx)
corr.re <- as.data.frame(bc.mssa) %>%
  select(pctnhw, pctnhb, pctnhai, pctnhapi, pcthisp, pct100pov, n_rate, early0iia)
corr.matrix <- na.omit(corr.re) 

# compute matrix of p-values to add to matrix
cor.mtest <- function(mat, ...) {
  mat <- as.matrix(mat)
  n <- ncol(mat)
  p.mat <- matrix(NA, n, n)
  diag(p.mat) <- 0
  for (i in 1:(n - 1)) {
    for (j in (i + 1): n) {
      tmp <- cor.test(mat[, i], mat[, j], ...)
      p.mat[i, j] <- p.mat[j, i] <- tmp$p.value}
  }
  colnames(p.mat) <- rownames(p.mat) <- colnames(mat)
  p.mat}

# matrix of the p-values of correlation
p.mat <- cor.mtest(corr.matrix)
head(p.mat[, 1:5])
```

```
##                 pctnhw       pctnhb     pctnhai     pctnhapi      pcthisp
## pctnhw    0.000000e+00 6.706055e-08 0.009822606 4.828049e-08 1.275566e-23
## pctnhb    6.706055e-08 0.000000e+00 0.005254443 2.990508e-19 9.654982e-01
## pctnhai   9.822606e-03 5.254443e-03 0.000000000 2.369855e-03 2.283547e-01
## pctnhapi  4.828049e-08 2.990508e-19 0.002369855 0.000000e+00 7.116027e-01
## pcthisp   1.275566e-23 9.654982e-01 0.228354665 7.116027e-01 0.000000e+00
## pct100pov 6.021721e-07 1.017497e-01 0.803361547 3.393363e-01 2.920780e-07
```

```
# correlation matrix - race/ethnicity, case rate, pov with corr coef & sig
corrplot(cor(corr.matrix), method = "color", type = "upper",
         order = "hclust", addCoef.col = "black", # add coef. of correlation
         tl.col = "black", tl.srt = 45, # text label color and rotation
         p.mat = p.mat, sig.level = 0.01, insig = "blank", # add significance)
         diag = FALSE) # hide correlation coef. on principal diagonal
```

2. GLM

```
# back up original values
bc.mssa <- bc.mssa %>%
           mutate(rural = ifelse(is.na(rural), "Urban", rural))
bc.mssa$rural2 = bc.mssa$rural
bc.mssa$pcsa2 = bc.mssa$pcsa

# factor binary variables
bc.mssa$rural <- as.factor(ifelse(bc.mssa$rural2 == "Rural", 1, 0))
bc.mssa$frontier <- as.factor(ifelse(bc.mssa$rural2 == "Frontier", 1, 0))
bc.mssa$pcsa <- as.factor(ifelse(bc.mssa$pcsa2 == 'Yes', 1, 0))

# subset for regression
reg.sub <- bc.mssa %>%
          mutate(percent_nhapi_nhaa = (pctnhapi + pctnhb),
                 percent_early = (early0iia/100),
                 n_early = round((percent_early*n_cases)),
                 # divide by 10 to make the change 10% increments
                 percent_poverty = (pct100pov/10),
                 percent_nhwhite = (pctnhw/10),
                 percent_hispanic = (pcthisp/10),
                 percent_nhapi_nhaa = (percent_nhapi_nhaa/10)) %>%
          select(mssa, percent_poverty, percent_nhwhite, 
                 percent_hispanic,
                 n_early,
                 female_pop = ftot, 
                 rural, frontier, percent_early,
                 primarycare_shortage = pcsa, 
                 percent_nhapi_nhaa, n_cases,
                 geometry)

# remove two MSSAs with suppressed dependent variable  
na.rm <- na.omit(reg.sub)

# 1. % early = % pov 
poverty <- glm(formula = cbind(n_early, n_cases) ~ percent_poverty + 
                 log(female_pop), 
               data = na.rm,
               family = "binomial")
summary(poverty)
```

```
## 
## Call:
## glm(formula = cbind(n_early, n_cases) ~ percent_poverty + log(female_pop), 
##     family = "binomial", data = na.rm)
## 
## Deviance Residuals: 
##      Min        1Q    Median        3Q       Max  
## -0.97949  -0.34086  -0.00244   0.27131   1.14135  
## 
## Coefficients:
##                   Estimate Std. Error z value Pr(>|z|)   
## (Intercept)     -0.2197199  0.1577837  -1.393  0.16376   
## percent_poverty -0.0425105  0.0143958  -2.953  0.00315 **
## log(female_pop)  0.0005661  0.0158718   0.036  0.97155   
## ---
## Signif. codes:  0 '***' 0.001 '**' 0.01 '*' 0.05 '.' 0.1 ' ' 1
## 
## (Dispersion parameter for binomial family taken to be 1)
## 
##     Null deviance: 30.179  on 94  degrees of freedom
## Residual deviance: 21.412  on 92  degrees of freedom
## AIC: 582.98
## 
## Number of Fisher Scoring iterations: 3
```

Negative coefficient = negative association between % residents
living in poverty in the MSSA and the percent of cases diagnosed early
in the MSSA. Percent poverty beta coefficient exponentiated is 0.958,
which is the change in odds ratio for a ten-percent increase in
area-level poverty.

```
# 2. % early = % pov + % nhw
nhwhite <- glm(formula = cbind(n_early, n_cases) ~ percent_poverty + 
                 percent_nhwhite + log(female_pop),
               data = na.rm,
               family = "binomial")
summary(nhwhite)
```

```
## 
## Call:
## glm(formula = cbind(n_early, n_cases) ~ percent_poverty + percent_nhwhite + 
##     log(female_pop), family = "binomial", data = na.rm)
## 
## Deviance Residuals: 
##      Min        1Q    Median        3Q       Max  
## -1.13442  -0.38543   0.02073   0.32078   0.87980  
## 
## Coefficients:
##                  Estimate Std. Error z value Pr(>|z|)  
## (Intercept)     -0.285533   0.169564  -1.684   0.0922 .
## percent_poverty -0.033516   0.016710  -2.006   0.0449 *
## percent_nhwhite  0.006233   0.005877   1.061   0.2889  
## log(female_pop)  0.002287   0.015955   0.143   0.8860  
## ---
## Signif. codes:  0 '***' 0.001 '**' 0.01 '*' 0.05 '.' 0.1 ' ' 1
## 
## (Dispersion parameter for binomial family taken to be 1)
## 
##     Null deviance: 30.179  on 94  degrees of freedom
## Residual deviance: 20.287  on 91  degrees of freedom
## AIC: 583.86
## 
## Number of Fisher Scoring iterations: 3
```

```
library(lmtest)
```

```
## Loading required package: zoo
```

```
## 
## Attaching package: 'zoo'
```

```
## The following objects are masked from 'package:base':
## 
##     as.Date, as.Date.numeric
```

```
lrtest(poverty, nhwhite)
```

```
## Likelihood ratio test
## 
## Model 1: cbind(n_early, n_cases) ~ percent_poverty + log(female_pop)
## Model 2: cbind(n_early, n_cases) ~ percent_poverty + percent_nhwhite + 
##     log(female_pop)
##   #Df  LogLik Df  Chisq Pr(>Chisq)
## 1   3 -288.49                     
## 2   4 -287.93  1 1.1247     0.2889
```

Percent non-Hispanic white not statistically significant. Attenuated
the effect of poverty slightly, but does not improve model fit.

```
# 3. % early = % pov + % hisp
hispanic <- glm(formula = cbind(n_early, n_cases) ~ percent_poverty + percent_hispanic + 
                  log(female_pop),
               data = na.rm,
               family = "binomial")
summary(hispanic)
```

```
## 
## Call:
## glm(formula = cbind(n_early, n_cases) ~ percent_poverty + percent_hispanic + 
##     log(female_pop), family = "binomial", data = na.rm)
## 
## Deviance Residuals: 
##      Min        1Q    Median        3Q       Max  
## -1.17466  -0.36129   0.01701   0.32234   0.92761  
## 
## Coefficients:
##                   Estimate Std. Error z value Pr(>|z|)  
## (Intercept)      -0.144614   0.163684  -0.883   0.3770  
## percent_poverty  -0.025230   0.017558  -1.437   0.1507  
## percent_hispanic -0.015695   0.009139  -1.717   0.0859 .
## log(female_pop)  -0.006042   0.016327  -0.370   0.7113  
## ---
## Signif. codes:  0 '***' 0.001 '**' 0.01 '*' 0.05 '.' 0.1 ' ' 1
## 
## (Dispersion parameter for binomial family taken to be 1)
## 
##     Null deviance: 30.179  on 94  degrees of freedom
## Residual deviance: 18.461  on 91  degrees of freedom
## AIC: 582.03
## 
## Number of Fisher Scoring iterations: 3
```

```
lrtest(poverty, hispanic)
```

```
## Likelihood ratio test
## 
## Model 1: cbind(n_early, n_cases) ~ percent_poverty + log(female_pop)
## Model 2: cbind(n_early, n_cases) ~ percent_poverty + percent_hispanic + 
##     log(female_pop)
##   #Df  LogLik Df  Chisq Pr(>Chisq)  
## 1   3 -288.49                       
## 2   4 -287.02  1 2.9502    0.08587 .
## ---
## Signif. codes:  0 '***' 0.001 '**' 0.01 '*' 0.05 '.' 0.1 ' ' 1
```

Percent poverty no longer significant when percent Hispanic is
included in the model. Percent Hispanic is not statistically significant
and does not improve model fit.

```
# 4. % early = % pov + (% nh asian/pacific islander or % nh african american/black)
# center data
api_aa <- glm(formula = cbind(n_early, n_cases) ~ percent_poverty + 
                percent_nhapi_nhaa + log(female_pop),
             data = na.rm,
             family = "binomial")
summary(api_aa)
```

```
## 
## Call:
## glm(formula = cbind(n_early, n_cases) ~ percent_poverty + percent_nhapi_nhaa + 
##     log(female_pop), family = "binomial", data = na.rm)
## 
## Deviance Residuals: 
##      Min        1Q    Median        3Q       Max  
## -0.98026  -0.34231  -0.00477   0.27321   1.13788  
## 
## Coefficients:
##                      Estimate Std. Error z value Pr(>|z|)   
## (Intercept)        -0.2205942  0.1638750  -1.346   0.1783   
## percent_poverty    -0.0424511  0.0147069  -2.886   0.0039 **
## percent_nhapi_nhaa -0.0001718  0.0086967  -0.020   0.9842   
## log(female_pop)     0.0006756  0.0168117   0.040   0.9679   
## ---
## Signif. codes:  0 '***' 0.001 '**' 0.01 '*' 0.05 '.' 0.1 ' ' 1
## 
## (Dispersion parameter for binomial family taken to be 1)
## 
##     Null deviance: 30.179  on 94  degrees of freedom
## Residual deviance: 21.411  on 91  degrees of freedom
## AIC: 584.98
## 
## Number of Fisher Scoring iterations: 3
```

```
lrtest(poverty, api_aa)
```

```
## Likelihood ratio test
## 
## Model 1: cbind(n_early, n_cases) ~ percent_poverty + log(female_pop)
## Model 2: cbind(n_early, n_cases) ~ percent_poverty + percent_nhapi_nhaa + 
##     log(female_pop)
##   #Df  LogLik Df Chisq Pr(>Chisq)
## 1   3 -288.49                    
## 2   4 -288.49  1 4e-04     0.9842
```

Poverty still statistically significant after NH-Asian/Pacific Island
or NH-African American/Black entered into the mode. The percent
NH-Asian/Pacific Islander or NH-African American/Black not statistically
significant and does not change beta coefficient for poverty.

```
# 6. % early = %pov rural/frontier
rural <- glm(formula = cbind(n_early, n_cases) ~ percent_poverty + 
               rural + frontier + log(female_pop),
            data = na.rm,
            family = "binomial")
summary(rural)
```

```
## 
## Call:
## glm(formula = cbind(n_early, n_cases) ~ percent_poverty + rural + 
##     frontier + log(female_pop), family = "binomial", data = na.rm)
## 
## Deviance Residuals: 
##      Min        1Q    Median        3Q       Max  
## -0.98477  -0.31203   0.00018   0.31075   1.13719  
## 
## Coefficients:
##                  Estimate Std. Error z value Pr(>|z|)   
## (Intercept)     -0.202182   0.231348  -0.874  0.38216   
## percent_poverty -0.042605   0.014472  -2.944  0.00324 **
## rural1          -0.001358   0.031338  -0.043  0.96545   
## frontier1       -0.034550   0.146063  -0.237  0.81301   
## log(female_pop) -0.001159   0.022685  -0.051  0.95926   
## ---
## Signif. codes:  0 '***' 0.001 '**' 0.01 '*' 0.05 '.' 0.1 ' ' 1
## 
## (Dispersion parameter for binomial family taken to be 1)
## 
##     Null deviance: 30.179  on 94  degrees of freedom
## Residual deviance: 21.355  on 90  degrees of freedom
## AIC: 586.93
## 
## Number of Fisher Scoring iterations: 3
```

```
lrtest(poverty, rural)
```

```
## Likelihood ratio test
## 
## Model 1: cbind(n_early, n_cases) ~ percent_poverty + log(female_pop)
## Model 2: cbind(n_early, n_cases) ~ percent_poverty + rural + frontier + 
##     log(female_pop)
##   #Df  LogLik Df  Chisq Pr(>Chisq)
## 1   3 -288.49                     
## 2   5 -288.46  2 0.0569     0.9719
```

Percent poverty still statistically significant and not attenuated
much with the inclusion of rural or frontier status entered into the
model. Rural (yes/no) and Frontier (yes/no) not statistically
significant (also tried them individually in case both were splitting
explanatory power and neither were significant on their own). No
improvement to model fit.

```
# 7. % late = %pov pcsa
pcsa <- glm(formula = cbind(n_early, n_cases) ~ percent_poverty +  primarycare_shortage + 
              log(female_pop),
           data = na.rm,
           family = "binomial")
summary(pcsa)
```

```
## 
## Call:
## glm(formula = cbind(n_early, n_cases) ~ percent_poverty + primarycare_shortage + 
##     log(female_pop), family = "binomial", data = na.rm)
## 
## Deviance Residuals: 
##     Min       1Q   Median       3Q      Max  
## -1.0489  -0.3675  -0.0284   0.2459   1.0326  
## 
## Coefficients:
##                        Estimate Std. Error z value Pr(>|z|)  
## (Intercept)           -0.179031   0.169982  -1.053   0.2922  
## percent_poverty       -0.036486   0.017173  -2.125   0.0336 *
## primarycare_shortage1 -0.017398   0.027035  -0.644   0.5199  
## log(female_pop)       -0.003921   0.017336  -0.226   0.8211  
## ---
## Signif. codes:  0 '***' 0.001 '**' 0.01 '*' 0.05 '.' 0.1 ' ' 1
## 
## (Dispersion parameter for binomial family taken to be 1)
## 
##     Null deviance: 30.179  on 94  degrees of freedom
## Residual deviance: 20.997  on 91  degrees of freedom
## AIC: 584.57
## 
## Number of Fisher Scoring iterations: 3
```

```
lrtest(poverty, pcsa)
```

```
## Likelihood ratio test
## 
## Model 1: cbind(n_early, n_cases) ~ percent_poverty + log(female_pop)
## Model 2: cbind(n_early, n_cases) ~ percent_poverty + primarycare_shortage + 
##     log(female_pop)
##   #Df  LogLik Df  Chisq Pr(>Chisq)
## 1   3 -288.49                     
## 2   4 -288.28  1 0.4142     0.5198
```

An MSSA being a primary care shortage area is not statistically
significantly associated with the % of cases diagnosed early cases in
the MSSA. Does not affect beta coefficient of poverty or improve model
fit.

```
# 8. % late = %pov, %hispanic, rural/frontier, pcsa
full <- glm(formula = cbind(n_early, n_cases) ~ percent_poverty + percent_hispanic + 
              rural + frontier +  primarycare_shortage + log(female_pop),
           data = na.rm,
           family = "binomial")
summary(full)
```

```
## 
## Call:
## glm(formula = cbind(n_early, n_cases) ~ percent_poverty + percent_hispanic + 
##     rural + frontier + primarycare_shortage + log(female_pop), 
##     family = "binomial", data = na.rm)
## 
## Deviance Residuals: 
##      Min        1Q    Median        3Q       Max  
## -1.23580  -0.31646   0.01391   0.39011   0.89492  
## 
## Coefficients:
##                        Estimate Std. Error z value Pr(>|z|)  
## (Intercept)           -0.048141   0.245851  -0.196   0.8448  
## percent_poverty       -0.020323   0.019691  -1.032   0.3020  
## percent_hispanic      -0.016344   0.009384  -1.742   0.0816 .
## rural1                -0.007792   0.032247  -0.242   0.8091  
## frontier1             -0.078071   0.148544  -0.526   0.5992  
## primarycare_shortage1 -0.013360   0.027516  -0.486   0.6273  
## log(female_pop)       -0.015794   0.024084  -0.656   0.5120  
## ---
## Signif. codes:  0 '***' 0.001 '**' 0.01 '*' 0.05 '.' 0.1 ' ' 1
## 
## (Dispersion parameter for binomial family taken to be 1)
## 
##     Null deviance: 30.179  on 94  degrees of freedom
## Residual deviance: 17.897  on 88  degrees of freedom
## AIC: 587.47
## 
## Number of Fisher Scoring iterations: 3
```

```
lrtest(poverty, full)
```

```
## Likelihood ratio test
## 
## Model 1: cbind(n_early, n_cases) ~ percent_poverty + log(female_pop)
## Model 2: cbind(n_early, n_cases) ~ percent_poverty + percent_hispanic + 
##     rural + frontier + primarycare_shortage + log(female_pop)
##   #Df  LogLik Df  Chisq Pr(>Chisq)
## 1   3 -288.49                     
## 2   7 -286.73  4 3.5143     0.4757
```

```
stargazer(poverty, nhwhite, hispanic, api_aa, rural, pcsa, full, type = "html", title = "Comparing Regression Results - poverty, poverty with NH-white, poverty with Hispanic, poverty with percent NH-Asian/Pacific Islander and NH-African American/Black, poverty with rural and frontier indicator, poverty with primary care shortage area indicator, full model with: poverty, percent Hispanic, rural and frontier indicator, primary care shortage area indicator")
```

**Comparing Regression Results - poverty, poverty with NH-white,
poverty with Hispanic, poverty with percent NH-Asian/Pacific Islander
and NH-African American/Black, poverty with rural and frontier
indicator, poverty with primary care shortage area indicator, full model
with: poverty, percent Hispanic, rural and frontier indicator, primary
care shortage area indicator**

|  | | | | | | | |
|  | *Dependent variable:* | | | | | | |
|  |  | | | | | | |
|  | cbind(n\_early, n\_cases) | | | | | | |
|  | (1) | (2) | (3) | (4) | (5) | (6) | (7) |
|  | | | | | | | |
| percent\_poverty | -0.043\*\*\* | -0.034\*\* | -0.025 | -0.042\*\*\* | -0.043\*\*\* | -0.036\*\* | -0.020 |
|  | (0.014) | (0.017) | (0.018) | (0.015) | (0.014) | (0.017) | (0.020) |
|  |  |  |  |  |  |  |  |
| percent\_nhwhite |  | 0.006 |  |  |  |  |  |
|  |  | (0.006) |  |  |  |  |  |
|  |  |  |  |  |  |  |  |
| percent\_hispanic |  |  | -0.016\* |  |  |  | -0.016\* |
|  |  |  | (0.009) |  |  |  | (0.009) |
|  |  |  |  |  |  |  |  |
| percent\_nhapi\_nhaa |  |  |  | -0.0002 |  |  |  |
|  |  |  |  | (0.009) |  |  |  |
|  |  |  |  |  |  |  |  |
| rural1 |  |  |  |  | -0.001 |  | -0.008 |
|  |  |  |  |  | (0.031) |  | (0.032) |
|  |  |  |  |  |  |  |  |
| frontier1 |  |  |  |  | -0.035 |  | -0.078 |
|  |  |  |  |  | (0.146) |  | (0.149) |
|  |  |  |  |  |  |  |  |
| primarycare\_shortage1 |  |  |  |  |  | -0.017 | -0.013 |
|  |  |  |  |  |  | (0.027) | (0.028) |
|  |  |  |  |  |  |  |  |
| log(female\_pop) | 0.001 | 0.002 | -0.006 | 0.001 | -0.001 | -0.004 | -0.016 |
|  | (0.016) | (0.016) | (0.016) | (0.017) | (0.023) | (0.017) | (0.024) |
|  |  |  |  |  |  |  |  |
| Constant | -0.220 | -0.286\* | -0.145 | -0.221 | -0.202 | -0.179 | -0.048 |
|  | (0.158) | (0.170) | (0.164) | (0.164) | (0.231) | (0.170) | (0.246) |
|  |  |  |  |  |  |  |  |
|  | | | | | | | |
| Observations | 95 | 95 | 95 | 95 | 95 | 95 | 95 |
| Log Likelihood | -288.491 | -287.929 | -287.016 | -288.491 | -288.463 | -288.284 | -286.734 |
| Akaike Inf. Crit. | 582.982 | 583.857 | 582.032 | 584.982 | 586.925 | 584.568 | 587.468 |
|  | | | | | | | |
| *Note:* | p<0.1; p<0.05; p<0.01 | | | | | | |

### Exponentiated parameter and confidence intervals

```
exp(cbind(OR = coef(poverty), confint(poverty)))
```

```
## Waiting for profiling to be done...
```

```
##                        OR     2.5 %    97.5 %
## (Intercept)     0.8027436 0.5890298 1.0933668
## percent_poverty 0.9583804 0.9317021 0.9857918
## log(female_pop) 1.0005663 0.9699442 1.0322115
```

```
exp(cbind(OR = coef(nhwhite), confint(nhwhite)))
```

```
## Waiting for profiling to be done...
```

```
##                        OR     2.5 %    97.5 %
## (Intercept)     0.7516133 0.5389330 1.0476512
## percent_poverty 0.9670397 0.9358726 0.9992261
## percent_nhwhite 1.0062526 0.9947277 1.0179114
## log(female_pop) 1.0022899 0.9714562 1.0341589
```

```
exp(cbind(OR = coef(hispanic), confint(hispanic)))
```

```
## Waiting for profiling to be done...
```

```
##                         OR     2.5 %   97.5 %
## (Intercept)      0.8653566 0.6276751 1.192365
## percent_poverty  0.9750859 0.9420848 1.009211
## percent_hispanic 0.9844279 0.9669456 1.002216
## log(female_pop)  0.9939763 0.9626960 1.026328
```

```
exp(cbind(OR = coef(api_aa), confint(api_aa)))
```

```
## Waiting for profiling to be done...
```

```
##                           OR     2.5 %    97.5 %
## (Intercept)        0.8020421 0.5815307 1.1055311
## percent_poverty    0.9584374 0.9311917 0.9864541
## percent_nhapi_nhaa 0.9998282 0.9829227 1.0170096
## log(female_pop)    1.0006758 0.9682641 1.0342267
```

```
exp(cbind(OR = coef(rural), confint(rural)))
```

```
## Waiting for profiling to be done...
```

```
##                        OR     2.5 %    97.5 %
## (Intercept)     0.8169459 0.5189754 1.2853427
## percent_poverty 0.9582899 0.9314765 0.9858468
## rural1          0.9986433 0.9391215 1.0618754
## frontier1       0.9660401 0.7242561 1.2847090
## log(female_pop) 0.9988420 0.9554251 1.0442826
```

```
exp(cbind(OR = coef(pcsa), confint(pcsa)))
```

```
## Waiting for profiling to be done...
```

```
##                              OR     2.5 %    97.5 %
## (Intercept)           0.8360803 0.5990285 1.1663883
## percent_poverty       0.9641720 0.9322526 0.9971723
## primarycare_shortage1 0.9827522 0.9320200 1.0362170
## log(female_pop)       0.9960870 0.9628298 1.0305374
```

```
exp(cbind(OR = coef(full), confint(full)))
```

```
## Waiting for profiling to be done...
```

```
##                              OR     2.5 %   97.5 %
## (Intercept)           0.9529990 0.5884349 1.542634
## percent_poverty       0.9798823 0.9427736 1.018429
## percent_hispanic      0.9837891 0.9658556 1.002045
## rural1                0.9922379 0.9314373 1.056945
## frontier1             0.9248990 0.6900866 1.236032
## primarycare_shortage1 0.9867287 0.9349092 1.041392
## log(female_pop)       0.9843301 0.9389662 1.031937
```

3. Diagnostics

```
library(ggfortify)

autoplot(poverty, which = 1, ncol = 1)
```

```
# goodness of fit
X = poverty %>%
  resid(type = "pearson")

chisq_stat = sum(X^2)

pval = pchisq(
  chisq_stat,
  lower = FALSE,
  df = length(X) - length(coef(poverty)))
```

```
# diagnostic plots
poverty %>%
  autoplot(which = 1:6)
```

4. Spatial Regression

```
fit.ols.simple <- lm(percent_early ~ percent_poverty + log(female_pop), data = na.rm)
summary(fit.ols.simple)
```

```
## 
## Call:
## lm(formula = percent_early ~ percent_poverty + log(female_pop), 
##     data = na.rm)
## 
## Residuals:
##      Min       1Q   Median       3Q      Max 
## -0.15472 -0.03535  0.00084  0.03000  0.14464 
## 
## Coefficients:
##                  Estimate Std. Error t value Pr(>|t|)    
## (Intercept)      0.841899   0.046628  18.055  < 2e-16 ***
## percent_poverty -0.024121   0.008208  -2.939  0.00417 ** 
## log(female_pop) -0.005435   0.005082  -1.069  0.28766    
## ---
## Signif. codes:  0 '***' 0.001 '**' 0.01 '*' 0.05 '.' 0.1 ' ' 1
## 
## Residual standard error: 0.05617 on 92 degrees of freedom
## Multiple R-squared:  0.09938,    Adjusted R-squared:  0.07981 
## F-statistic: 5.076 on 2 and 92 DF,  p-value: 0.008106
```

```
# map residuals from simple regression to see if visual evidence of autocorrelation in error
na.rm <- na.rm %>%
            mutate(olsresid = resid(fit.ols.simple))

# plot residuals
tm_shape(na.rm) +
    tm_polygons(col = "olsresid", style = "equal", palette = "Reds", border.alpha = 0, 
                title = "") +
  tm_scale_bar(breaks = c(0, 2, 4), text.size = 1, position = c("right", "bottom")) +
  tm_layout(main.title = "Residuals from simple linear regression in California MSSAs", main.title.size = 0.95, frame = FALSE, legend.outside = TRUE, attr.outside = TRUE)
```

There may be some clustering of the residual, but unclear from visual
detection

```
# spatial autocorrelation
#### using Queen contiguity with row-standardized weights

# neighbors
mssab <- poly2nb(na.rm, queen = TRUE)

# weights
mssaw <- nb2listw(mssab, style = "W", zero.policy = TRUE)

# Moran scatterplot
moran.plot(na.rm$percent_early, listw = mssaw, 
           xlab = "Standardized Percent Early Diagnosis", 
           ylab = "Neighbors Standardized Percent Early Diagnosis", 
           main = c("Moran Scatterplot for Early Breast Cancer Diagnoses", 
                    "in California MSSAs"))
```

```
# calculate Global Moran's I using monte carlo simulation to get the p-value
moran.mc(na.rm$percent_early, mssaw, nsim = 999)
```

```
## 
##  Monte-Carlo simulation of Moran I
## 
## data:  na.rm$percent_early 
## weights: mssaw  
## number of simulations + 1: 1000 
## 
## statistic = 0.14686, observed rank = 987, p-value = 0.013
## alternative hypothesis: greater
```

```
# repeat for the OLS residuals using moran test
lm.morantest(poverty, mssaw)
```

```
## 
##  Global Moran I for regression residuals
## 
## data:  
## model: glm(formula = cbind(n_early, n_cases) ~ percent_poverty +
## log(female_pop), family = "binomial", data = na.rm)
## weights: mssaw
## 
## Moran I statistic standard deviate = 2.7013, p-value = 0.003454
## alternative hypothesis: greater
## sample estimates:
## Observed Moran I      Expectation         Variance 
##      0.131507083     -0.071276581      0.005635384
```

Does not appear to be a strong association based on the Moran
scatterplot, maybe a slightly positive one as indicated by the line of
best fit. Global Moran’s I is statistically significant - it is possible
there is spatial autocorrelation in the model. However, Moran’s I for
the residuals from the OLS regression with percent poverty only
statistically significant at the p < 0.1 level

```
# spatial lag model - models spatial dependency in the outcome
spatial_lag <- lagsarlm(percent_early ~ percent_poverty + 
                      log(female_pop), data = na.rm, listw = mssaw)
summary(spatial_lag)
```

```
## 
## Call:lagsarlm(formula = percent_early ~ percent_poverty + log(female_pop), 
##     data = na.rm, listw = mssaw)
## 
## Residuals:
##         Min          1Q      Median          3Q         Max 
## -0.15424174 -0.03015879 -0.00018899  0.02959516  0.15626155 
## 
## Type: lag 
## Coefficients: (asymptotic standard errors) 
##                   Estimate Std. Error z value  Pr(>|z|)
## (Intercept)      0.6730454  0.1185983  5.6750 1.387e-08
## percent_poverty -0.0213568  0.0080453 -2.6546  0.007941
## log(female_pop) -0.0049956  0.0049242 -1.0145  0.310338
## 
## Rho: 0.21296, LR test value: 2.1937, p-value: 0.13858
## Asymptotic standard error: 0.14084
##     z-value: 1.5121, p-value: 0.13051
## Wald statistic: 2.2864, p-value: 0.13051
## 
## Log likelihood: 141.3646 for lag model
## ML residual variance (sigma squared): 0.0029584, (sigma: 0.054391)
## Number of observations: 95 
## Number of parameters estimated: 5 
## AIC: -272.73, (AIC for lm: -272.54)
## LM test for residual autocorrelation
## test value: 2.2402, p-value: 0.13447
```

Percent poverty continues to be statistically significant, Rho value
is not statistically significant, indicating any spatial lag in the
dependent variable is accounted for through the poverty variable already
included in the model

```
# spatial error model - models spatial dependency in the residuals
spatial_error <- errorsarlm(percent_early ~ percent_poverty + 
                        log(female_pop), data = na.rm, listw = mssaw)
summary(spatial_error)
```

```
## 
## Call:errorsarlm(formula = percent_early ~ percent_poverty + log(female_pop), 
##     data = na.rm, listw = mssaw)
## 
## Residuals:
##         Min          1Q      Median          3Q         Max 
## -0.15946745 -0.03311194 -0.00096338  0.02927974  0.15019463 
## 
## Type: error 
## Coefficients: (asymptotic standard errors) 
##                   Estimate Std. Error z value Pr(>|z|)
## (Intercept)      0.8351202  0.0477579 17.4865  < 2e-16
## percent_poverty -0.0216467  0.0084616 -2.5582  0.01052
## log(female_pop) -0.0051258  0.0052214 -0.9817  0.32625
## 
## Lambda: 0.16898, LR test value: 1.1235, p-value: 0.28917
## Asymptotic standard error: 0.14915
##     z-value: 1.1329, p-value: 0.25725
## Wald statistic: 1.2835, p-value: 0.25725
## 
## Log likelihood: 140.8295 for error model
## ML residual variance (sigma squared): 0.0030023, (sigma: 0.054793)
## Number of observations: 95 
## Number of parameters estimated: 5 
## AIC: -271.66, (AIC for lm: -272.54)
```

Percent poverty continues to be statistically significant. Lambda is
positive, but statistically insignificant. Indicating we do not need to
control for spatial autocorrelation in the error (our original
coefficient estimates and signs of the IV are correct, and the standard
errors are not underestimated)

```
# compare models
AICs <- c(AIC(fit.ols.simple), AIC(spatial_lag), AIC(spatial_error))
labels <- c("OOLS", "SLM", "SEM")

kable(data.frame(Models = labels, AIC = round(AICs, 2)))
```

| Models | AIC |
| --- | --- |
| OOLS | -272.54 |
| SLM | -272.73 |
| SEM | -271.66 |

```
# Lagrange Multiplier test (significance rejects OLS)
lm.LMtests(fit.ols.simple, listw = mssaw, test = "all", zero.policy = TRUE)
```

```
## 
##  Lagrange multiplier diagnostics for spatial dependence
## 
## data:  
## model: lm(formula = percent_early ~ percent_poverty + log(female_pop),
## data = na.rm)
## weights: mssaw
## 
## LMerr = 1.0923, df = 1, p-value = 0.296
## 
## 
##  Lagrange multiplier diagnostics for spatial dependence
## 
## data:  
## model: lm(formula = percent_early ~ percent_poverty + log(female_pop),
## data = na.rm)
## weights: mssaw
## 
## LMlag = 2.4062, df = 1, p-value = 0.1209
## 
## 
##  Lagrange multiplier diagnostics for spatial dependence
## 
## data:  
## model: lm(formula = percent_early ~ percent_poverty + log(female_pop),
## data = na.rm)
## weights: mssaw
## 
## RLMerr = 2.7834, df = 1, p-value = 0.09524
## 
## 
##  Lagrange multiplier diagnostics for spatial dependence
## 
## data:  
## model: lm(formula = percent_early ~ percent_poverty + log(female_pop),
## data = na.rm)
## weights: mssaw
## 
## RLMlag = 4.0973, df = 1, p-value = 0.04295
## 
## 
##  Lagrange multiplier diagnostics for spatial dependence
## 
## data:  
## model: lm(formula = percent_early ~ percent_poverty + log(female_pop),
## data = na.rm)
## weights: mssaw
## 
## SARMA = 5.1896, df = 2, p-value = 0.07466
```

LMerr nor LMlag statistically significant. There does not appear to
be spatial autocorrelation and the spatial lag or spatial error model
does not seem appropriate.

```
stargazer(fit.ols.simple, type = "html", title = "Regression Results")
```

**Regression Results**

|  | |
|  | *Dependent variable:* |
|  |  |
|  | percent\_early |
|  | |
| percent\_poverty | -0.024\*\*\* |
|  | (0.008) |
|  |  |
| log(female\_pop) | -0.005 |
|  | (0.005) |
|  |  |
| Constant | 0.842\*\*\* |
|  | (0.047) |
|  |  |
|  | |
| Observations | 95 |
| R2 | 0.099 |
| Adjusted R2 | 0.080 |
| Residual Std. Error | 0.056 (df = 92) |
| F Statistic | 5.076\*\*\* (df = 2; 92) |
|  | |
| *Note:* | p<0.1; p<0.05; p<0.01 |
